# Supplementary material for: Alternative lengthening of telomeres in childhood neuroblastoma from genome to proteome
Source: Nat Commun. 2021 Feb 24;12:1269. doi: 10.1038/s41467-021-21247-8 (PMC7904810; doi:10.1038/s41467-021-21247-8)
Supplement: Supplementary file 1 — Supplementary Information [file 41467_2021_21247_MOESM1_ESM.pdf]

Supplementary Information:

Alternative lengthening of telomeres in childhood neuroblastoma from genome to proteome

Hartlieb and Sieverling et al.

I. Supplementary Figures .... - 2 -

Supplementary Figure 1: Telomere content and C-Circle intensity of discovery and INFORM cohort - 2 -
Supplementary Figure 2: C-Circle status of discovery and INFORM cohort .... - 4 -
Supplementary Figure 3: Silencing of *TERT* locus, telomerase activity and raw TERRA counts .... - 6 -
Supplementary Figure 4: Comparison of matching primary and relapse pairs .... - 7 -
Supplementary Figure 5: Combined *MYCN* and telomere FISH .... - 8 -
Supplementary Figure 6: Most frequent differentially mutated genes in ALT-positive tumors .... - 10 -
Supplementary Figure 7: *CDK4/CCND1* expression and mutations in Telnet genes .... - 12 -
Supplementary Figure 8: Overall copy number patterns of ALT-positive tumors compared to ALT negative tumors of the discovery cohort. .... - 13 -
Supplementary Figure 9: *SYNE1* and *PTPRD* .... - 15 -
Supplementary Figure 10: Mutational landscape of INFORM cohort .... - 16 -
Supplementary Figure 11: Comparison of mRNA and protein data .... - 17 -
Supplementary Figure 12: Exon specific expression of *ATRX* and most frequent mutations in ALT-positive *ATRX* wild-type neuroblastomas .... - 20 -
Supplementary Figure 13: Mutations in ATRX/DAXX interaction partners .... - 22 -
Supplementary Figure 14: Chromatin state at SatII and SatIII sequences .... - 23 -
Supplementary Figure 15: Features of telomeric repeat loci .... - 26 -
Supplementary Figure 16: 1q42.2-1qter deletions .... - 27 -

II. Supplementary Tables .... - 28 -

Supplementary Table 1: Two-sided telomeric repeat loci .... - 28 -
Supplementary Table 2: siRNA sequences .... - 28 -
Supplementary Table 3: Antibodies .... - 28 -
Supplementary Table 4: DEXseq *ATRX* exon model .... - 29 -

III. Supplementary References .... - 30 -

I. Supplementary Figures

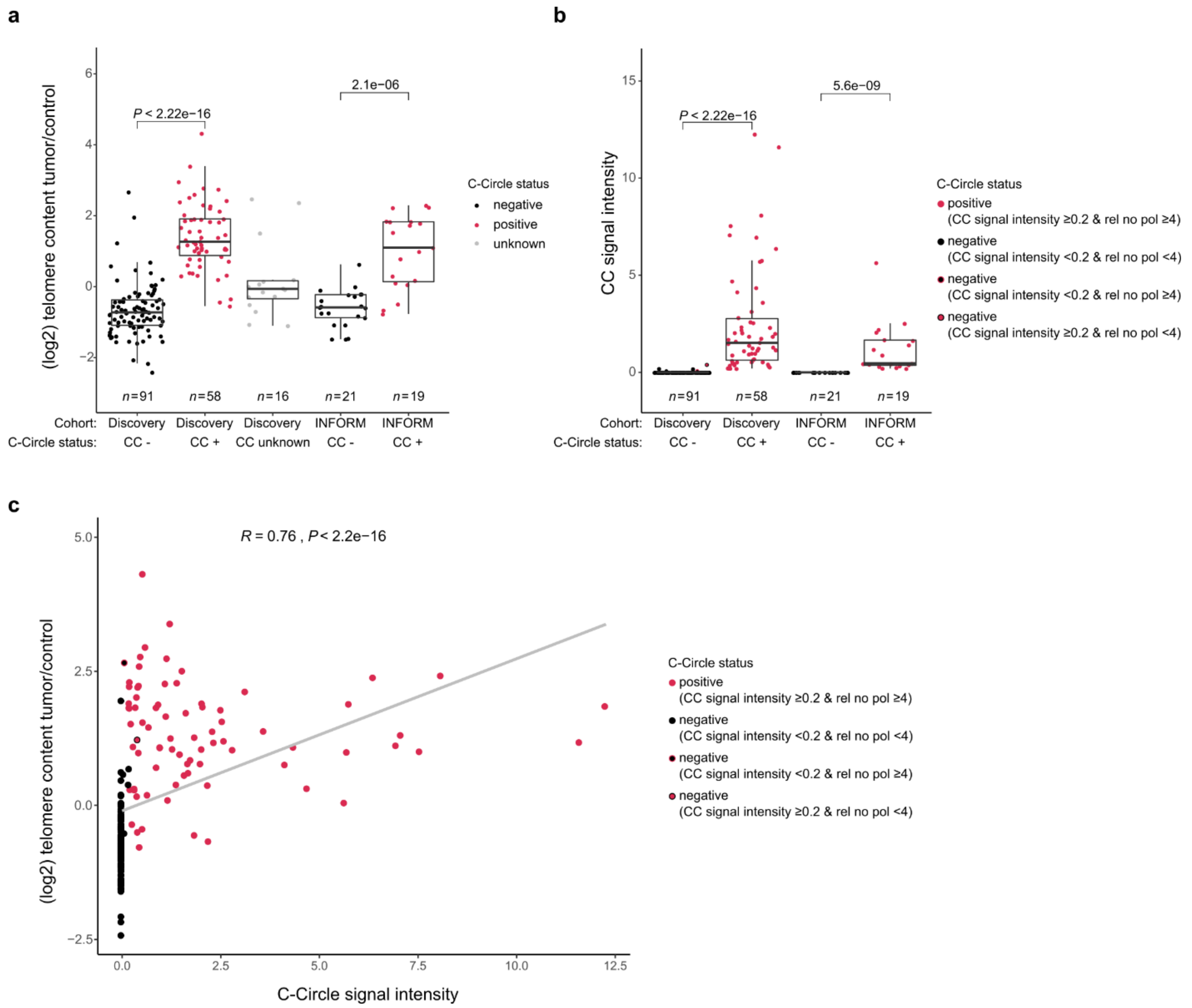

Supplementary Figure 1: Telomere content and C-Circle intensity of discovery and INFORM cohort

(a) Relative telomere content of C-Circle (CC) positive and negative tumors in the discovery and INFORM cohort. (b) C-Circle intensity relative to CHLA-90 positive control for tumors in the discovery and INFORM cohort. Color-coding indicates the C-Circle status using the two applied criteria of a signal intensity relative to CHLA-90 positive control ≥0.2 and a signal intensity relative to the negative control without polymerase ≥4. Due to the high number of samples, not all tumors could be analyzed on the same blot. Thus, every blot contained a CHLA-90 positive control and signal intensities were normalized to this control. *(a-b)* Boxplots indicate the median value (middle line) and the 25th and 75th percentiles (box). The upper/lower whisker spans from the hinge to the largest/smallest value (values expanding a distance of 1.5 x inter quartile range are not considered). Dots represent individual tumors. *P* values were calculated using two-sided Wilcoxon rank sum tests. *n* describes the number of analyzed tumors. (c) Scatter plot of telomere content relative to C-Circle intensity. Color-coding indicates C-Circle status. Correlation coefficient was calculated using spearman correlation.

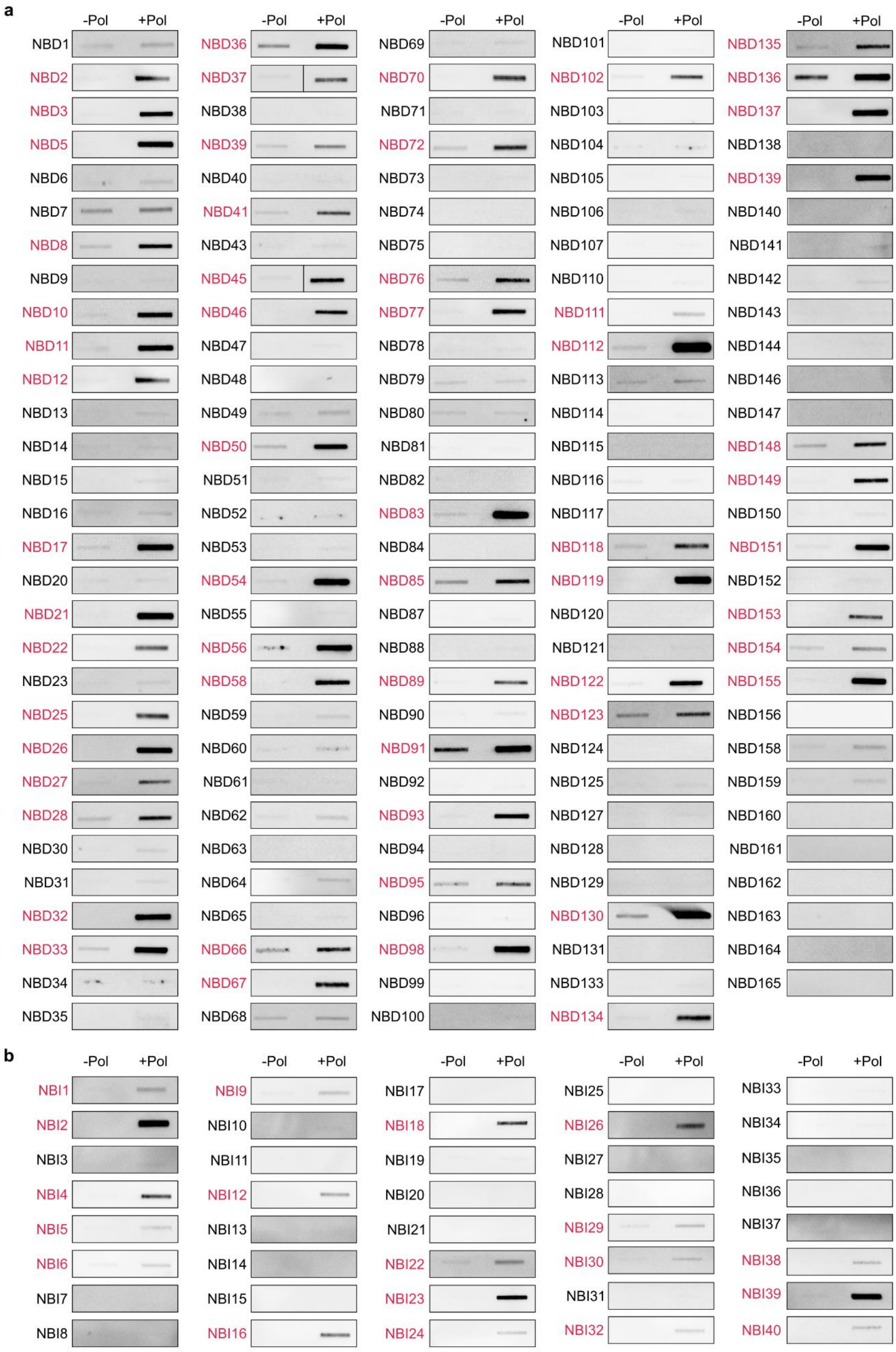
a
-Pol
+Pol
NBD1
NBD2
NBD3
NBD5
NBD6
NBD7
NBD8
NBD9
NBD10
NBD11
NBD12
NBD13
NBD14
NBD15
NBD16
NBD17
NBD20
NBD21
NBD22
NBD23
NBD25
NBD26
NBD27
NBD28
NBD30
NBD31
NBD32
NBD33
NBD34
NBD35
NBD36
NBD37
NBD38
NBD39
NBD40
NBD41
NBD43
NBD45
NBD46
NBD47
NBD48
NBD49
NBD50
NBD51
NBD52
NBD53
NBD54
NBD55
NBD56
NBD58
NBD59
NBD60
NBD61
NBD62
NBD63
NBD64
NBD65
NBD66
NBD67
NBD68
NBD69
NBD70
NBD71
NBD72
NBD73
NBD74
NBD75
NBD76
NBD77
NBD78
NBD79
NBD80
NBD81
NBD82
NBD83
NBD84
NBD85
NBD87
NBD88
NBD89
NBD90
NBD91
NBD92
NBD93
NBD94
NBD95
NBD96
NBD98
NBD99
NBD100
NBD101
NBD102
NBD103
NBD104
NBD105
NBD106
NBD107
NBD110
NBD111
NBD112
NBD113
NBD114
NBD115
NBD116
NBD117
NBD118
NBD119
NBD120
NBD121
NBD122
NBD123
NBD124
NBD125
NBD127
NBD128
NBD129
NBD130
NBD131
NBD133
NBD134
NBD135
NBD136
NBD137
NBD138
NBD139
NBD140
NBD141
NBD142
NBD143
NBD144
NBD146
NBD147
NBD148
NBD149
NBD150
NBD151
NBD152
NBD153
NBD154
NBD155
NBD156
NBD158
NBD159
NBD160
NBD161
NBD162
NBD163
NBD164
NBD165
b
-Pol
+Pol
NBI1
NBI2
NBI3
NBI4
NBI5
NBI6
NBI7
NBI8
NBI9
NBI10
NBI11
NBI12
NBI13
NBI14
NBI15
NBI16
NBI17
NBI18
NBI19
NBI20
NBI21
NBI22
NBI23
NBI24
NBI25
NBI26
NBI27
NBI28
NBI29
NBI30
NBI31
NBI32
NBI33
NBI34
NBI35
NBI36
NBI37
NBI38
NBI39
NBI40

Supplementary Figure 2: C-Circle status of discovery and INFORM cohort

C-Circle slot blot images of tumors of the discovery cohort (a) and INFORM cohort (b). Control without polymerase (-Pol) was analyzed for all samples. C-Circle positive tumors are marked in red. Due to the high number of samples, not all tumors could be analyzed on the same blot. Thus, every blot contained a CHLA-90 positive control. Every tumor was analyzed once. Uncut images are shown in source data file.

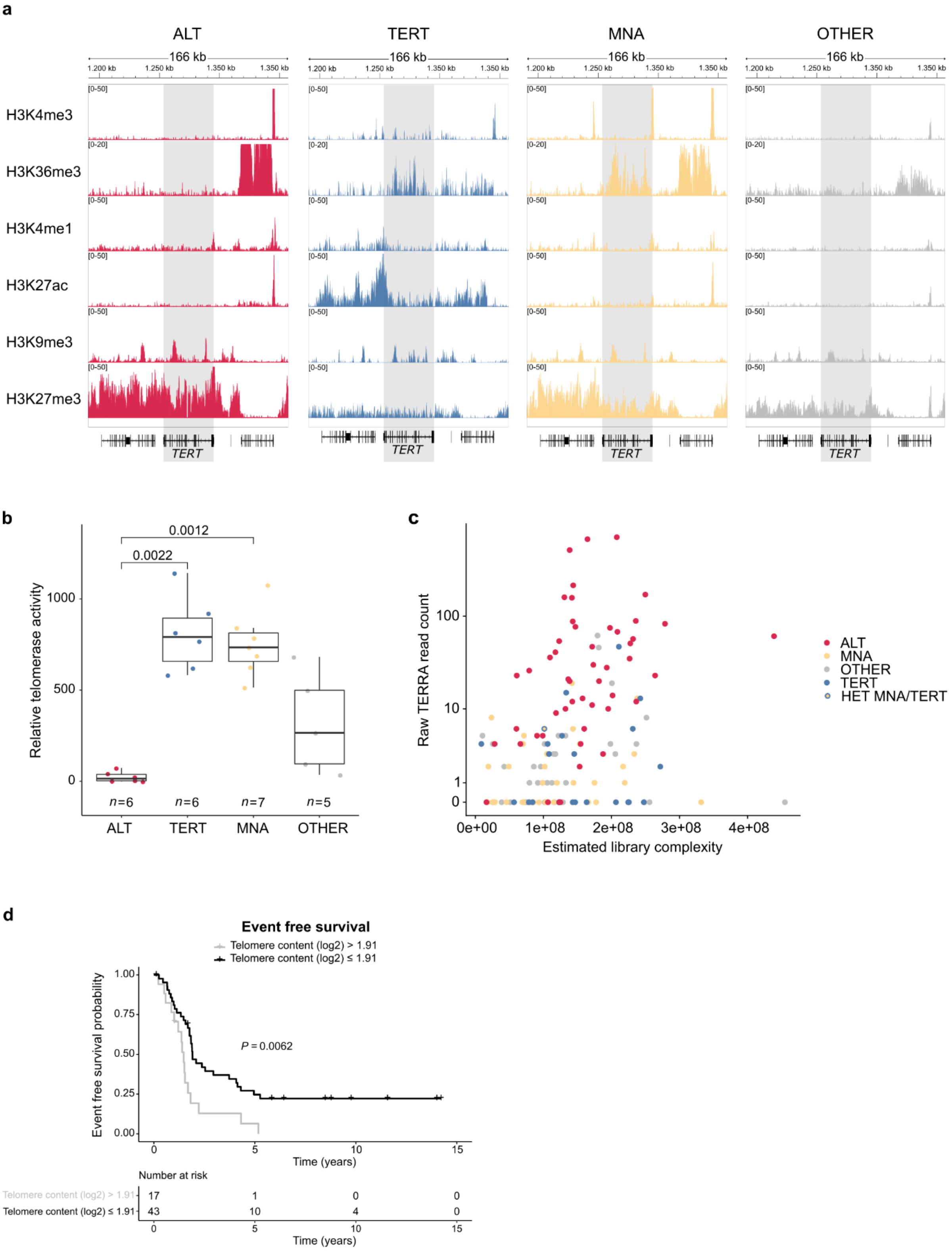
a
ALT
TERT
MNA
OTHER
166 kb
H3K4me3
H3K36me3
H3K4me1
H3K27ac
H3K9me3
H3K27me3
TERT
b
0.0012
0.0022
Relative telomerase activity
n=6
n=6
n=7
n=5
ALT
TERT
MNA
OTHER
c
Raw TERRA read count
Estimated library complexity
ALT
MNA
OTHER
TERT
HET MNA/TERT
d
Event free survival
Telomere content (log2) > 1.91
Telomere content (log2) ≤ 1.91
Event free survival probability
P = 0.0062
Time (years)
Number at risk
Telomere content (log2) > 1.91 17 1 0 0
Telomere content (log2) ≤ 1.91 43 10 4 0
Time (years)

Supplementary Figure 3: Silencing of *TERT* locus, telomerase activity and raw TERRA counts
(a) ChIP sequencing profiles of the *TERT* locus of exemplary tumor samples from all subgroups: ALT (red), TERT (blue), MNA (yellow) and OTHER (grey). (b) Relative telomerase activity in different neuroblastoma subgroups: ALT (red), TERT (blue), MNA (yellow) and OTHER (grey). Boxplots indicate the median value (middle line) and the 25th and 75th percentiles (box). The upper/lower whisker spans from the hinge to the largest/smallest value (values expanding a distance of 1.5 x inter quartile range are not considered). Dots represent individual tumors. *n* describes the number of analyzed tumors. *P*-values were calculated using a two-sided Wilcoxon rank sum test. (c) Number of raw TERRA read counts relative to the estimated library complexity. Color-coding indicates TMM group. (d) Event free survival of ALT-positive neuroblastomas in the discovery cohort. ALT-positive tumors are separated based on a very high (>1.91) telomere content. Cut point was calculated using Maximally Selected Rank Statistics. *P* value was calculated using a Log-rank test.

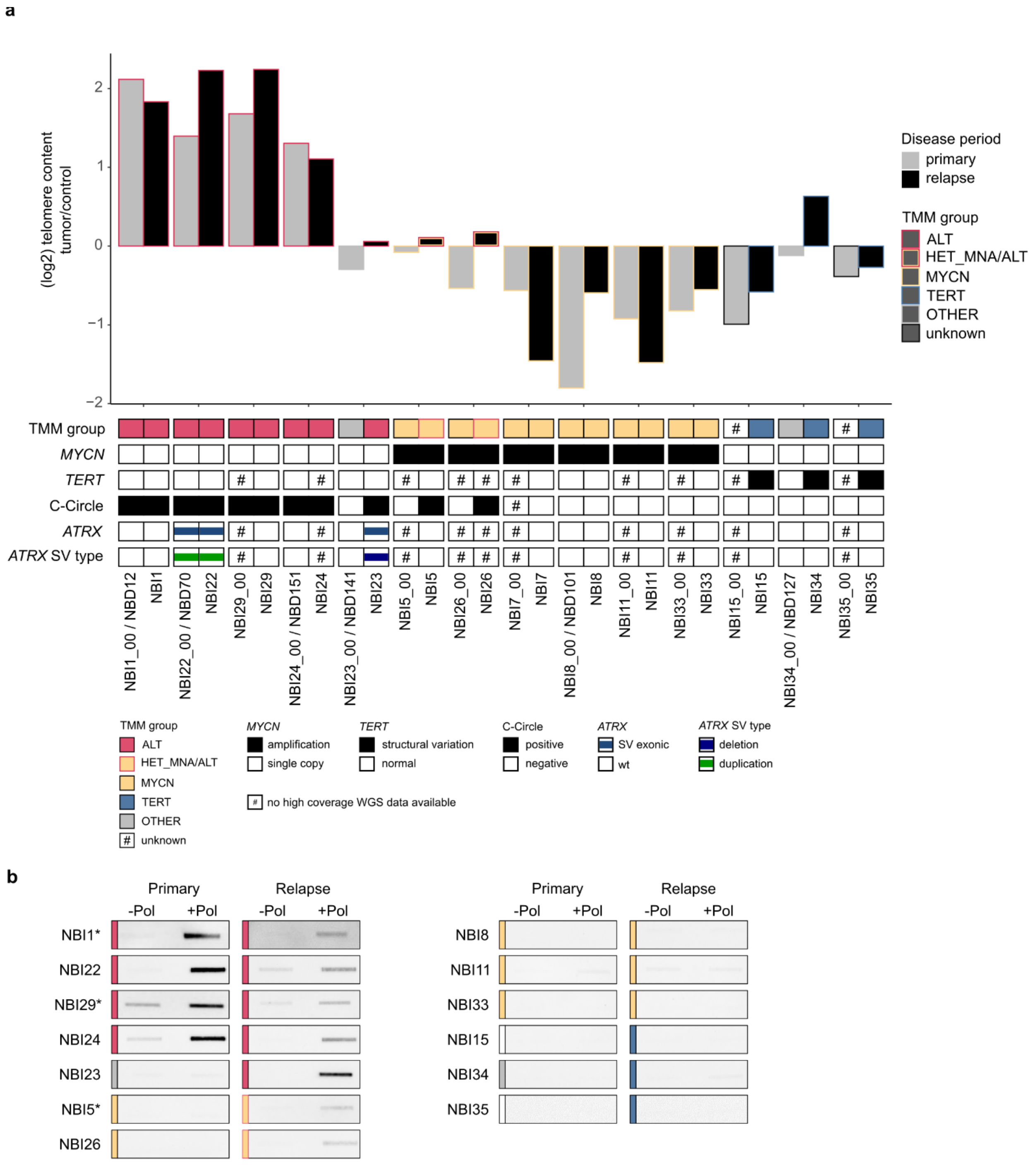

Supplementary Figure 4: Comparison of matching primary and relapse pairs

(a) Telomere content determined using lcWGS data of matching primary and relapse pairs. C-Circle status and *MYCN* status is given. For samples with available hcWGS data *TERT* status and *ATRX* mutation status is given. (b) C-Circle images of matching primary and relapse pairs. Color-coding on the left side indicates the TMM group (see legend a). For every tumor, a control without polymerase (-Pol) is shown. For samples labeled with * primary and relapse tumor were not analyzed on the same blot. Every tumor was analyzed once. Uncut images are given in source data file.

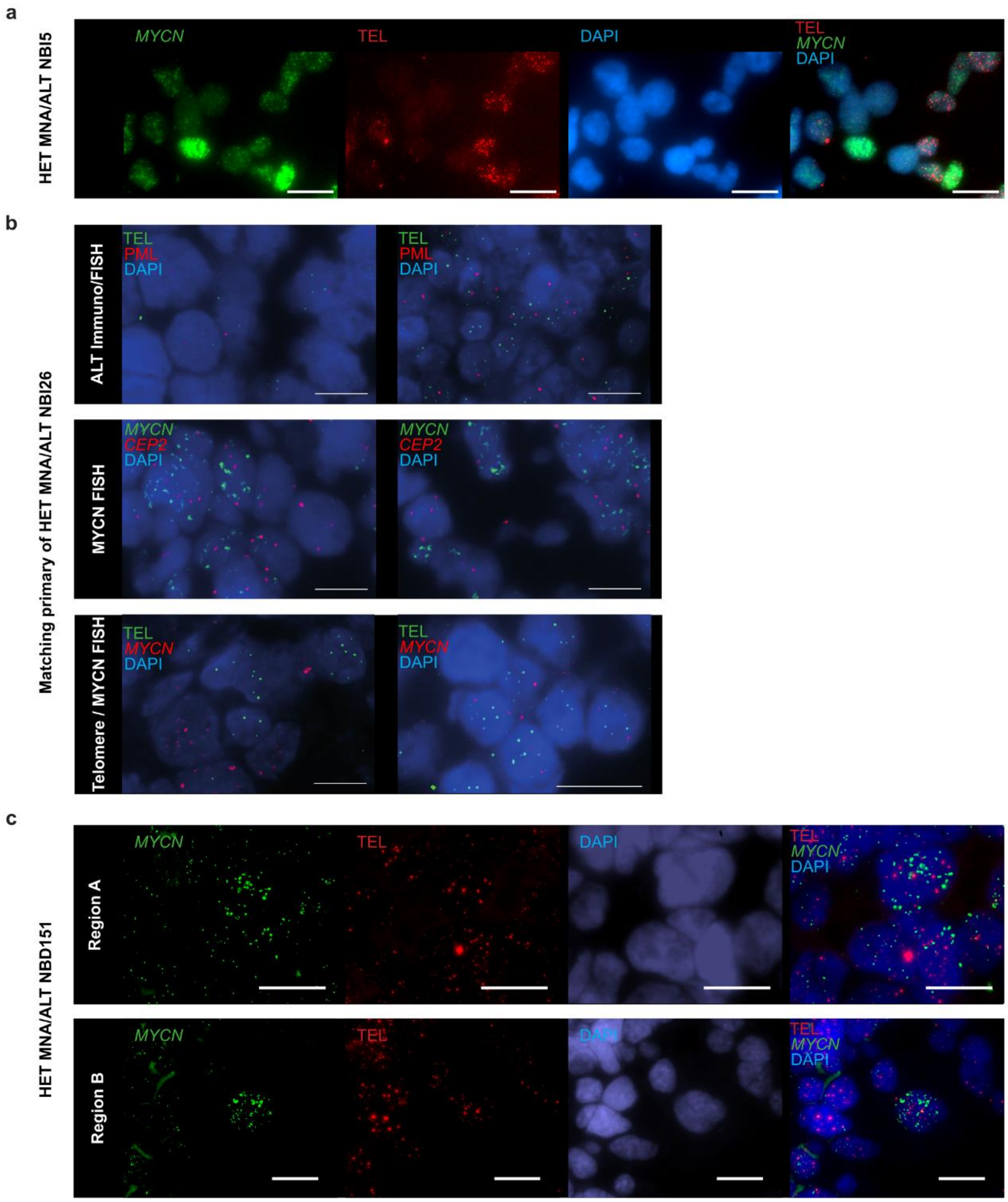

Supplementary Figure 5: Combined *MYCN* and telomere FISH

(a) *MYCN* and telomere FISH of HET MNA/ALT tumor NBI5. Additionally, DAPI staining and a merged image are shown. Scale bar representing 20 µm. (b) PML immunostaining and telomere FISH of the matching primary of HET MNA/ALT NBI26 (top panel). *MYCN* and *CEP2* FISH of the matching primary of HET MNA/ALT tumor NBI26 (middle panel). *MYCN* and telomere FISH of the matching primary of HET MNA/ALT tumor NBI26 (bottom panel). Scale bar representing 10 µm. (c) *MYCN* and telomere FISH of HET MNA/ALT tumor NBD151. Additionally, DAPI staining and a merged image are shown. Scale bar representing 20 µm. Every tumor was analyzed once.

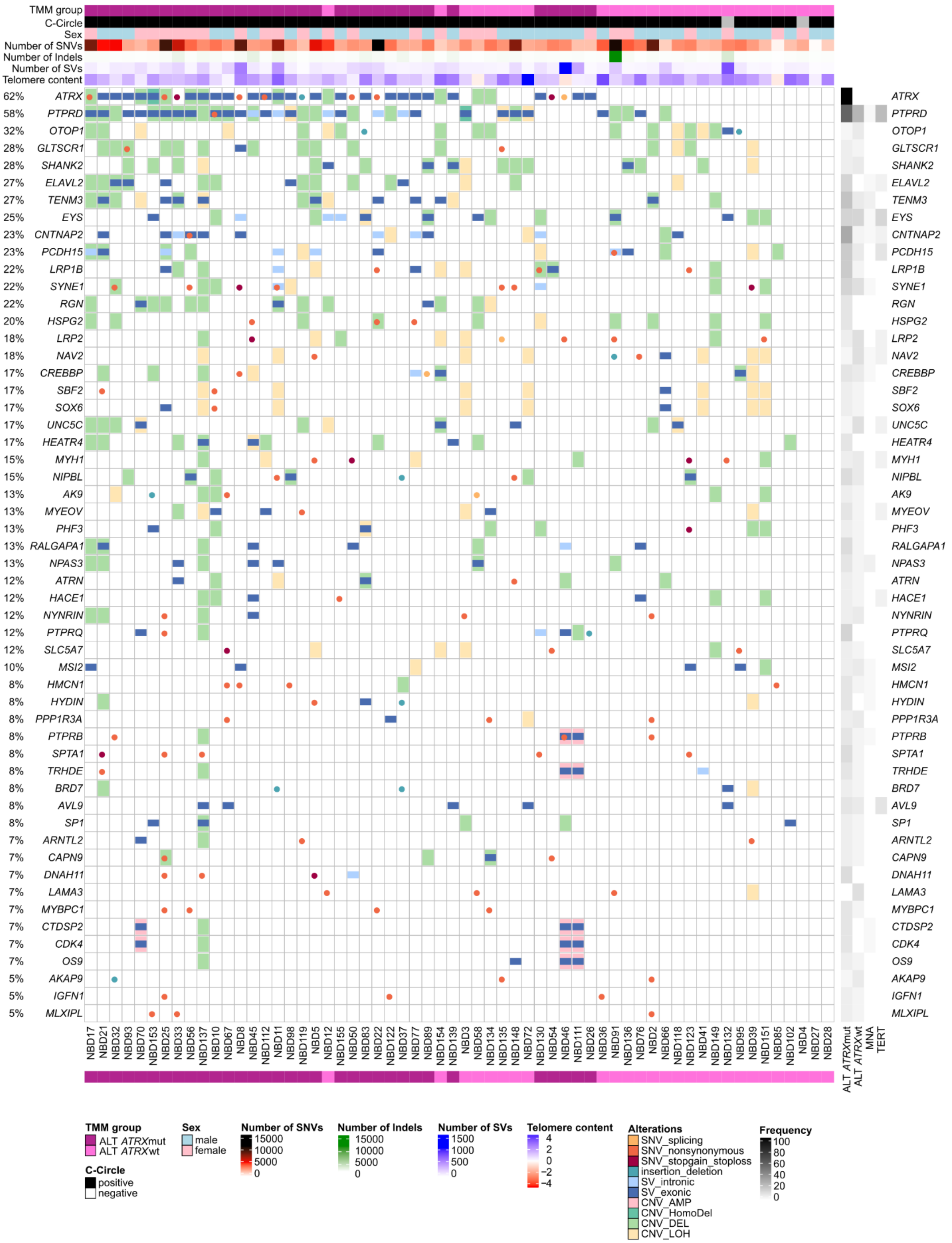
TMM group
C-Circle
Sex
Number of SNVs
Number of Indels
Number of SVs
Telomere content
62% ATRX
58% PTPRD
32% OTOP1
28% GLTSCR1
28% SHANK2
27% ELAVL2
27% TENM3
25% EYS
23% CNTNAP2
23% PCDH15
22% LRP1B
22% SYNE1
22% RGN
20% HSPG2
18% LRP2
18% NAV2
17% CREBBP
17% SBF2
17% SOX6
17% UNC5C
17% HEATR4
15% MYH1
15% NIPBL
13% AK9
13% MYEOV
13% PHF3
13% RALGAPA1
13% NPAS3
12% ATRN
12% HACE1
12% NYNRIN
12% PTPRQ
12% SLC5A7
10% MSI2
8% HMCN1
8% HYDIN
8% PPP1R3A
8% PTPRB
8% SPTA1
8% TRHDE
8% BRD7
8% AVL9
8% SP1
7% ARNTL2
7% CAPN9
7% DNAH11
7% LAMA3
7% MYBPC1
7% CTDSP2
7% CDK4
7% OS9
5% AKAP9
5% IGFN1
5% MLXIPL
NBD17 NBD21 NBD32 NBD93 NBD70 NBD153 NBD25 NBD33 NBD56 NBD137 NBD10 NBD67 NBD8 NBD45 NBD112 NBD11 NBD98 NBD119 NBD5 NBD12 NBD155 NBD50 NBD83 NBD22 NBD122 NBD37 NBD77 NBD89 NBD154 NBD139 NBD3 NBD58 NBD134 NBD135 NBD148 NBD72 NBD130 NBD54 NBD46 NBD111 NBD26 NBD36 NBD91 NBD136 NBD76 NBD2 NBD66 NBD118 NBD123 NBD41 NBD149 NBD132 NBD95 NBD39 NBD151 NBD85 NBD102 NBD4 NBD27 NBD28
ALT ATRXmut
ALT ATRXwt
MNA
TERT
TMM group
ALT ATRXmut
ALT ATRXwt
C-Circle
positive
negative
Sex
male
female
Number of SNVs
15000
10000
5000
0
Number of Indels
15000
10000
5000
0
Number of SVs
1500
1000
500
0
Telomere content
4
2
0
−2
−4
Alterations
SNV_splicing
SNV_nonsynonymous
SNV_stopgain_stoploss
insertion_deletion
SV_intronic
SV_exonic
CNV_AMP
CNV_HomoDel
CNV_DEL
CNV_LOH
Frequency
100
80
60
40
20
0

Supplementary Figure 6: Most frequent differentially mutated genes in ALT-positive tumors
Somatic alterations were identified in whole genome sequencing data of 60 ALT-positive samples. Exonic single nucleotide variations (SNVs), exonic small insertions and deletions (INDELs), structural variations in exonic and intronic regions (SVs) as well as copy number changes (CNVs) are illustrated. For detailed color-coding and symbols, see legend. See top panel for telomere maintenance features and total number of called SNVs, SVs and INDELs. Frequency calculations on the left are based on SNVs, SVs, INDELs and CNVs. Black and white heatmap on the right side indicates the frequency of mutations of the respective gene in neuroblastoma subgroups. Copy number changes (deletion, LOH) were not considered for frequency calculations in the heatmap (right panel).

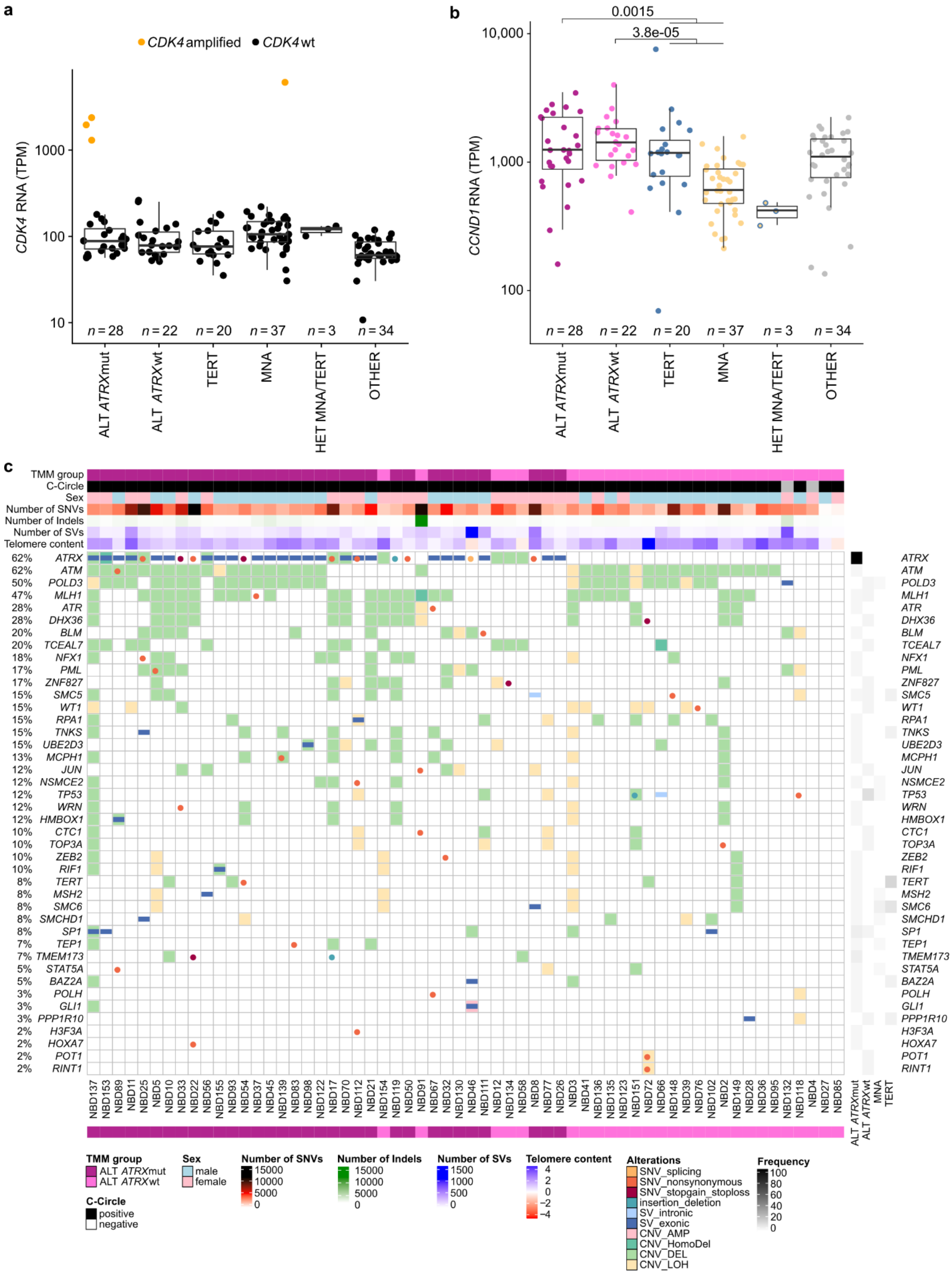

a
CDK4 amplified
CDK4 wt
CDK4 RNA (TPM)
1000
100
10
n = 28
n = 22
n = 20
n = 37
n = 3
n = 34
ALT ATRXmut
ALT ATRXwt
TERT
MNA
HET MNA/TERT
OTHER
b
0.0015
3.8e-05
10,000
1,000
100
CCND1 RNA (TPM)
n = 28
n = 22
n = 20
n = 37
n = 3
n = 34
ALT ATRXmut
ALT ATRXwt
TERT
MNA
HET MNA/TERT
OTHER
c
TMM group
C-Circle
Sex
Number of SNVs
Number of Indels
Number of SVs
Telomere content
62% ATRX
62% ATM
50% POLD3
47% MLH1
28% ATR
28% DHX36
20% BLM
20% TCEAL7
18% NFX1
17% PML
17% ZNF827
15% SMC5
15% WT1
15% RPA1
15% TNKS
15% UBE2D3
13% MCPH1
12% JUN
12% NSMCE2
12% TP53
12% WRN
12% HMBOX1
10% CTC1
10% TOP3A
10% ZEB2
10% RIF1
8% TERT
8% MSH2
8% SMC6
8% SMCHD1
8% SP1
7% TEP1
7% TMEM173
5% STAT5A
5% BAZ2A
3% POLH
3% GLI1
3% PPP1R10
2% H3F3A
2% HOXA7
2% POT1
2% RINT1
NBD137 NBD153 NBD89 NBD11 NBD25 NBD5 NBD10 NBD33 NBD22 NBD56 NBD155 NBD93 NBD54 NBD37 NBD45 NBD139 NBD83 NBD98 NBD122 NBD17 NBD70 NBD112 NBD21 NBD154 NBD119 NBD50 NBD91 NBD67 NBD32 NBD130 NBD46 NBD111 NBD12 NBD134 NBD58 NBD8 NBD77 NBD26 NBD3 NBD41 NBD136 NBD135 NBD123 NBD151 NBD72 NBD66 NBD148 NBD39 NBD76 NBD102 NBD2 NBD149 NBD28 NBD36 NBD95 NBD132 NBD118 NBD4 NBD27 NBD85
ALT ATRXmut
ALT ATRXwt
MNA
TERT
TMM group
ALT ATRXmut
ALT ATRXwt
C-Circle
positive
negative
Sex
male
female
Number of SNVs
15000
10000
5000
0
Number of Indels
15000
10000
5000
0
Number of SVs
1500
1000
500
0
Telomere content
4
2
0
−2
−4
Alterations
SNV_splicing
SNV_nonsynonymous
SNV_stopgain_stoploss
insertion_deletion
SV_intronic
SV_exonic
CNV_AMP
CNV_HomoDel
CNV_DEL
CNV_LOH
Frequency
100
80
60
40
20
0

Supplementary Figure 7: *CDK4/CCND1* expression and mutations in Telnet genes

(a) mRNA expression of *CDK4* in neuroblastoma subgroups. *CDK4* amplified tumors are highlighted. (b) mRNA expression of *CCND1* in neuroblastoma subgroups: ALT *ATRX*-mutated (dark pink), ALT *ATRX* wild-type (light pink), TERT (blue), MNA (yellow) and OTHER (grey) (a-b) Boxplots indicate the median value (middle line) and the 25th and 75th percentiles (box). The upper/lower whisker spans from the hinge to the largest/smallest value (values expanding a distance of 1.5 x inter quartile range are not considered). Dots represent individual tumors. *P* values were calculated with two-sided Wilcoxon rank sum tests. *n* describes the number of analyzed tumors. (c) Mutations in telomere maintenance associated genes in ALT-positive tumors. Somatic alterations were identified in whole genome sequencing data of 60 ALT-positive samples. Only genes reported in the TelNet database as validated to be involved in telomere maintenance[1] are shown. Exonic single nucleotide variations (SNVs), exonic small insertions and deletions (INDELs), structural variations in exonic and intronic regions (SVs) as well as copy number changes are illustrated. See top panel for telomere features and total number of called SNVs, SVs and INDELs. Frequency calculations on the left are based on SNVs, SVs, INDELs and CNVs. Black and white heatmap on the right side indicates the frequency of mutations in the respective gene in neuroblastoma groups of different telomere maintenance mechanisms. Copy number changes (deletion, LOH) were not considered for frequency calculations in the heatmap (right panel).

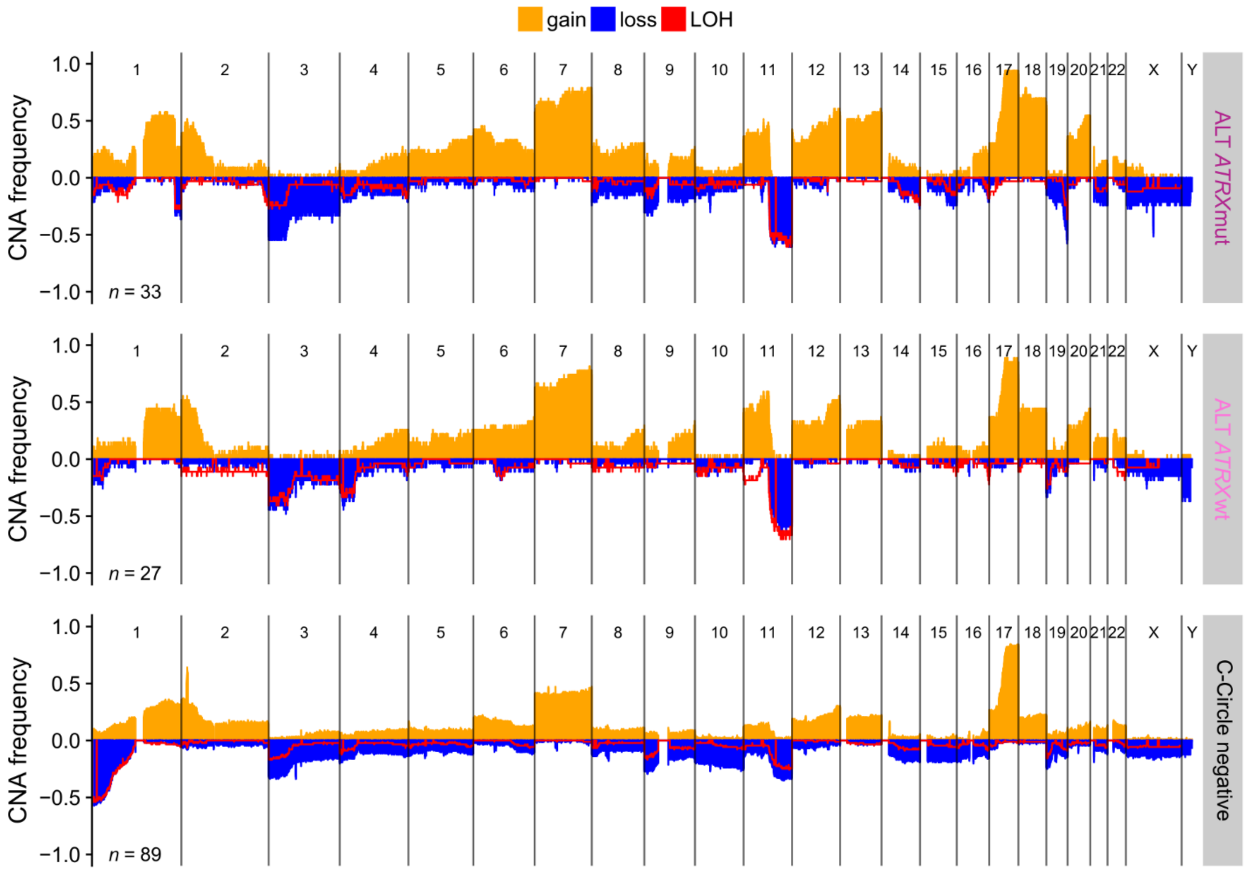

Supplementary Figure 8: Overall copy number patterns of ALT-positive tumors compared to ALT negative tumors of the discovery cohort.

CNV frequencies of ALT-positive tumors are further subdivided in *ATRX*-mutated and wild-type. Chromosomes are illustrated along the horizontal axis and frequencies of CNVs along the vertical axis. Gains are illustrated in yellow and losses in blue. LOH is shown as a red line. *n* represents the number of tumors in each group.

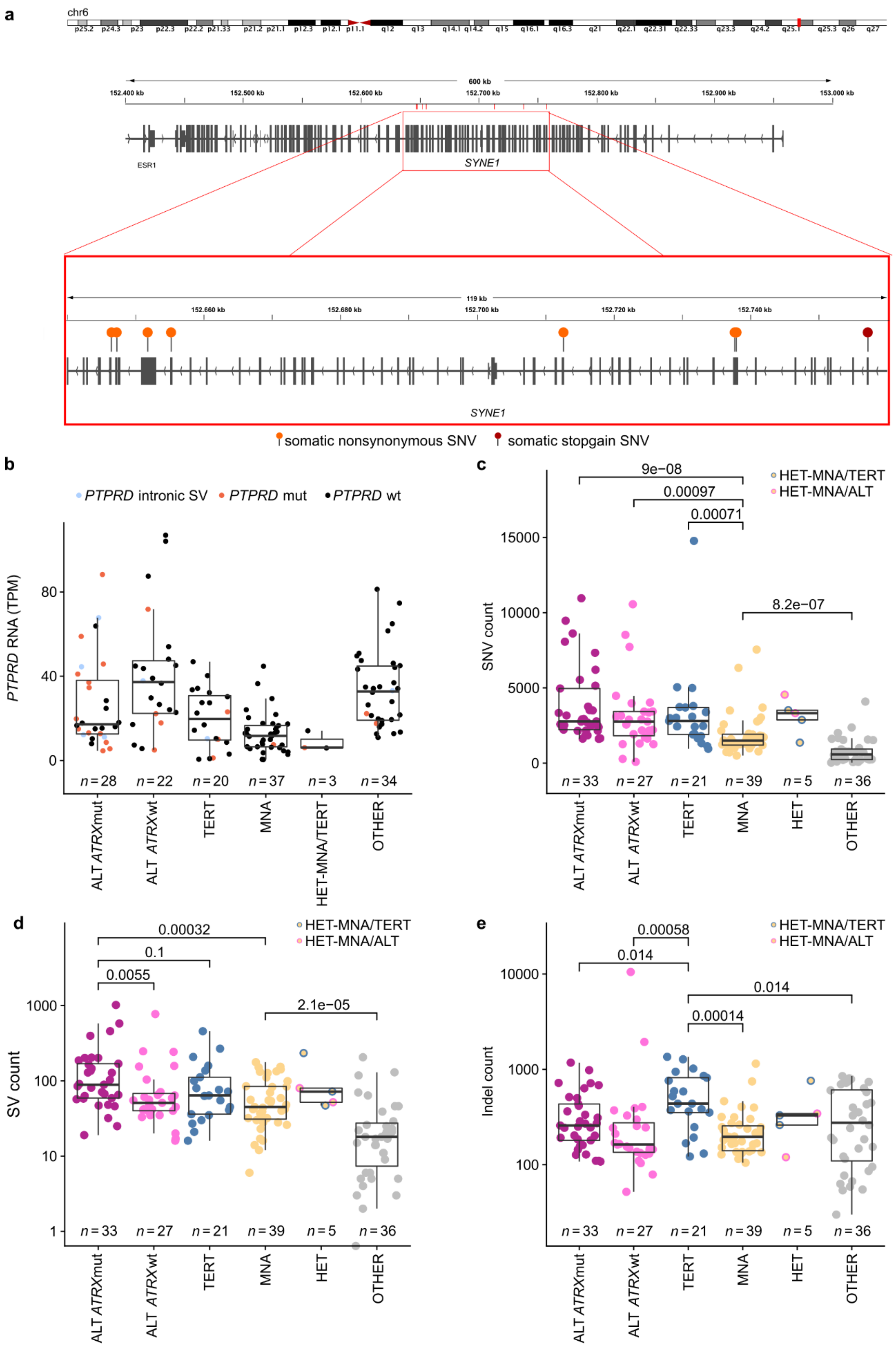
a
chr6
600 kb
SYNE1
ESR1
119 kb
somatic nonsynonymous SNV
somatic stopgain SNV
b
PTPRD intronic SV
PTPRD mut
PTPRD wt
PTPRD RNA (TPM)
n = 28
n = 22
n = 20
n = 37
n = 3
n = 34
ALT ATRXmut
ALT ATRXwt
TERT
MNA
HET-MNA/TERT
OTHER
c
9e−08
0.00097
0.00071
8.2e−07
HET-MNA/TERT
HET-MNA/ALT
SNV count
n = 33
n = 27
n = 21
n = 39
n = 5
n = 36
ALT ATRXmut
ALT ATRXwt
TERT
MNA
HET
OTHER
d
0.00032
0.1
0.0055
2.1e−05
HET-MNA/TERT
HET-MNA/ALT
SV count
e
0.00058
0.014
0.014
0.00014
HET-MNA/TERT
HET-MNA/ALT
Indel count

Supplementary Figure 9: *SYNE1* and *PTPRD*

(a) Exact genomic location of *SYNE1* SNVs. (b) mRNA expression of *PTPRD* in neuroblastoma subgroups. Tumors with *PTPRD* mutations are highlighted. Total number of SNVs (c), SVs (d) and INDELs (e) in the discovery cohort. Samples without a matching blood control sample were excluded from this analysis. (b-e) *P* values were calculated with two-sided Wilcoxon rank sum tests. Boxplots indicate the median value (middle line) and the 25th and 75th percentiles (box). The upper/lower whisker spans from the hinge to the largest/smallest value (values expanding a distance of 1.5 x inter quartile range are not considered). Dots represent individual tumors. *n* describes the number of analyzed tumors. Colors indicate TMM group: ALT *ATRX*-mutated (dark pink), ALT *ATRX* wild-type (light pink), TERT (blue), MNA (yellow) and OTHER (grey).

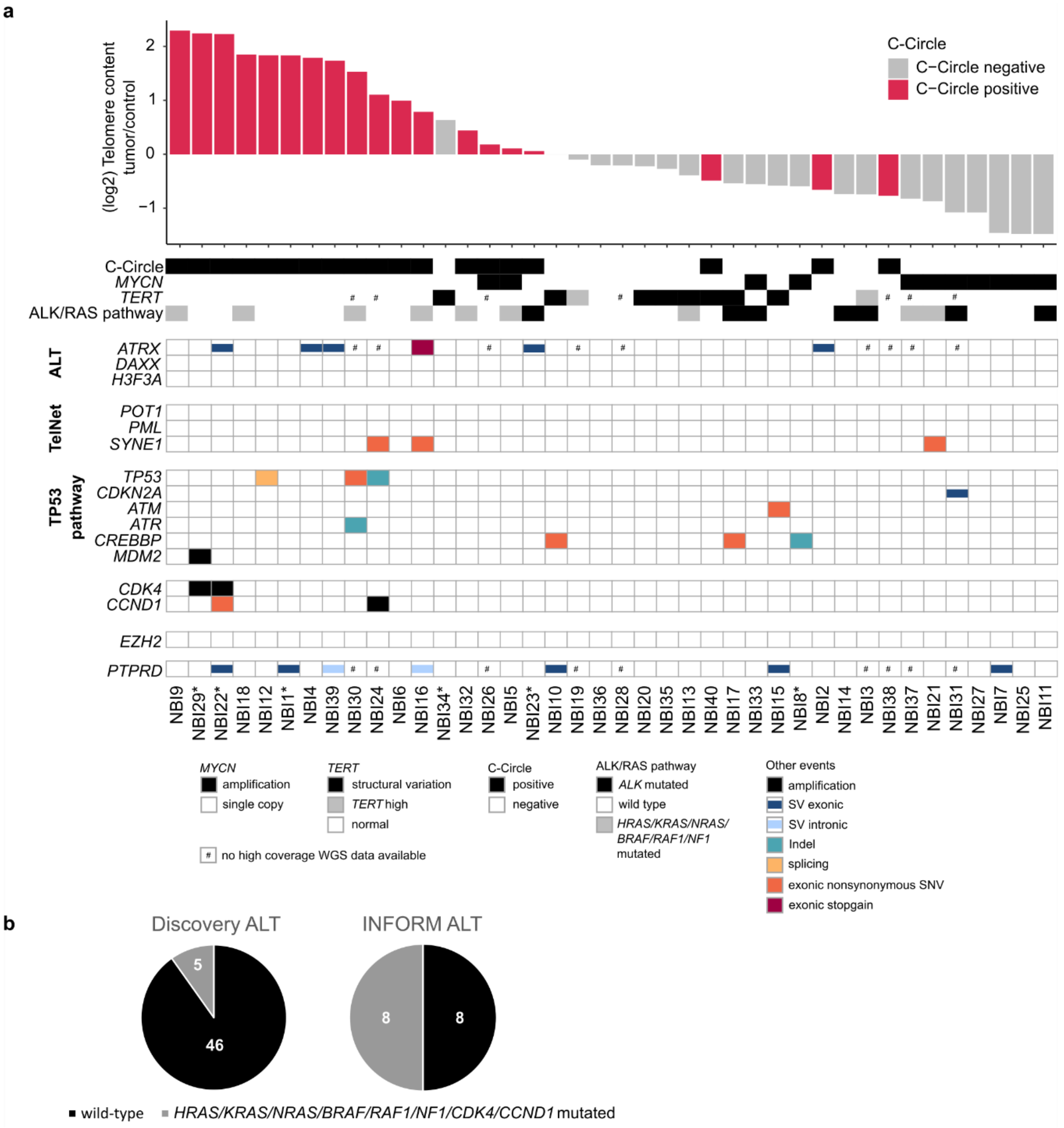

Supplementary Figure 10: Mutational landscape of INFORM cohort

(a) Telomere features and co-mutations in a cohort of relapsed neuroblastomas enrolled in the registry trial INFORM. The top panel indicates telomere maintenance mechanism, relative telomere content (tumor/control) and presence of ALK/RAS pathway mutations (*HRAS*, *NRAS*, *KRAS*, *BRAF*, *RAF1*, *NF1* or *ALK*). Co-mutations in ALT-associated genes, genes associated with telomere biology according to the TelNet database[1] and TP53 pathway genes are shown. Somatic co-mutations of relapsed patients were identified using WES and low coverage WGS data. *ATRX* mutations, *TERT* SVs and *PTPRD* SVs were analyzed using high coverage whole genome sequencing (# no high coverage WGS data was available). *) Sample is part of a matching primary relapse pair. (b) Frequency of RAS pathway mutations (*HRAS, KRAS, NRAS, BRAF, RAF1, NF1, CDK4, CCND1*) in ALT-positive neuroblastomas in the discovery and INFORM cohort. Relapse cases in the discovery cohort and HET cases in the INFORM cohort were excluded from the analysis.

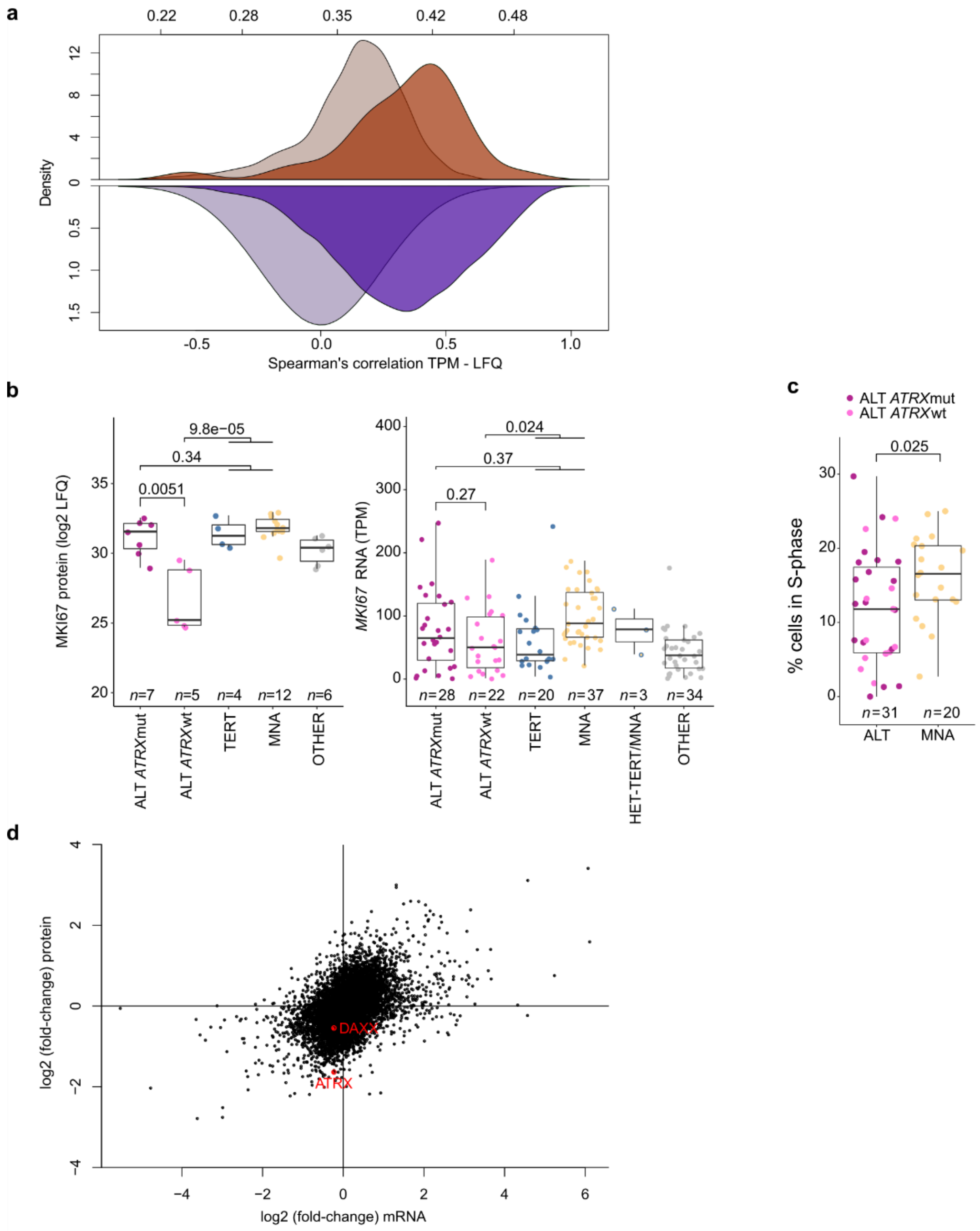

Supplementary Figure 11: Comparison of mRNA and protein data
(a) Distributions of Spearman's correlation coefficients between mRNA and protein abundance. (top) between different transcript-protein pairs per matching tumor sample (orange, mean Spearman's correlation coefficient = 0.4) and with random tumor sample (grey, mean Spearman's correlation coefficient = 0.36). (bottom) between varying abundances of transcript and proteins per matching genes (with at least 10 paired measurements) (purple, mean Spearman's correlation coefficient = 0.32) and with random gene (grey, mean Spearman's correlation coefficient = 0). (b) Protein and mRNA expression of *MKI67* in neuroblastoma tumors of the discovery cohort. (c) Percentage of cells in S-phase compared between ALT-positive and MNA tumors determined by FACS analysis of $n = 51$ cases. (b-c) Colors indicate TMM group: ALT *ATRX*-mutated (dark pink), ALT *ATRX* wild-type (light pink), TERT (blue), MNA (yellow) and OTHER (grey). Boxplots indicate the median value (middle line) and the 25th and 75th

percentiles (box). The upper/lower whisker spans from the hinge to the largest/smallest value (values expanding a distance of 1.5 x inter quartile range are not considered). Dots represent individual tumors. *P* values were calculated with two-sided Wilcoxon rank sum tests. *n* describes the number of analyzed tumors. (d) Comparison of mRNA and protein fold-changes between ALT tumors and neuroblastomas of the other TMM groups (MNA, TERT, OTHER).

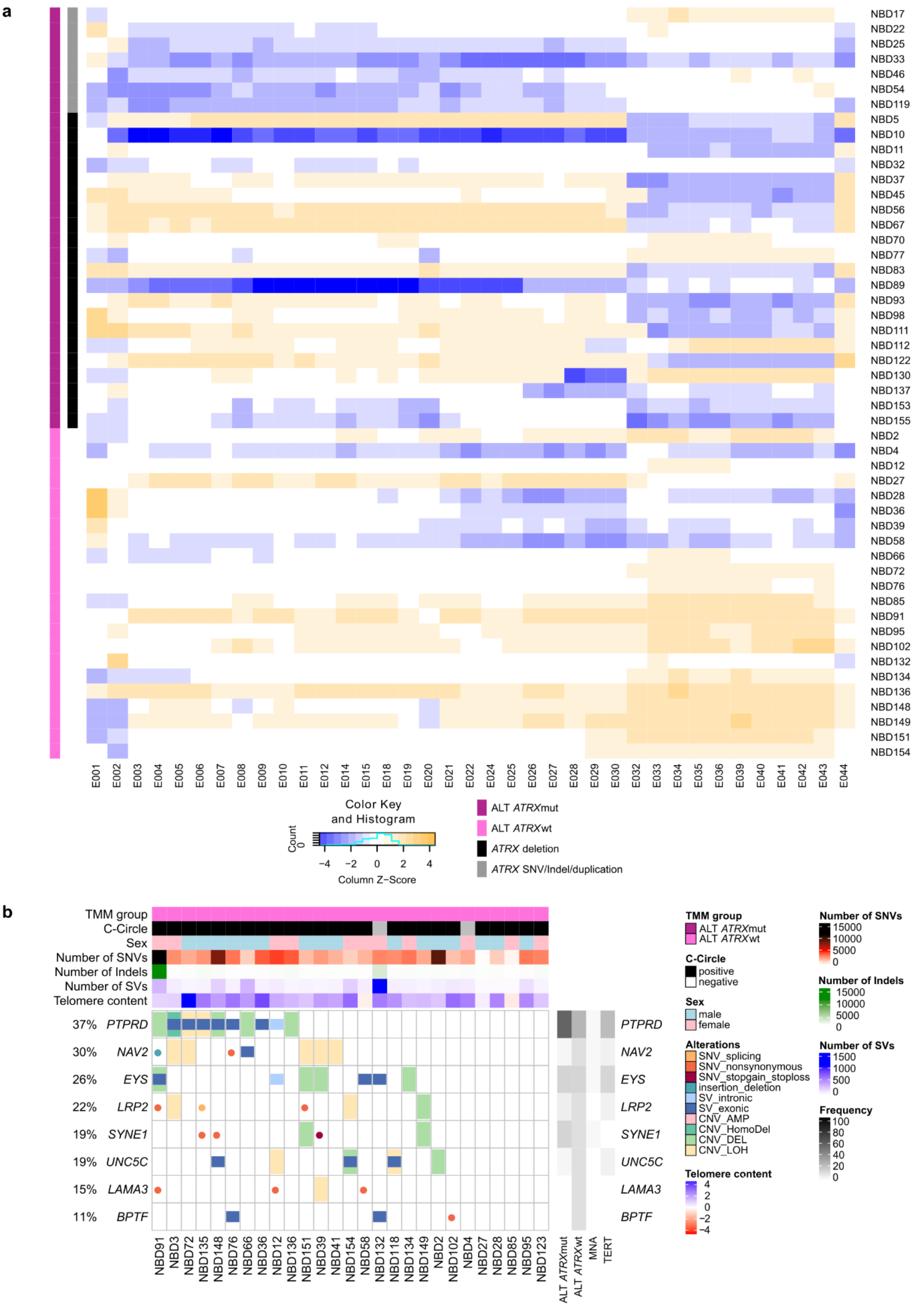
a
NBD17
NBD22
NBD25
NBD33
NBD46
NBD54
NBD119
NBD5
NBD10
NBD11
NBD32
NBD37
NBD45
NBD56
NBD67
NBD70
NBD77
NBD83
NBD89
NBD93
NBD98
NBD111
NBD112
NBD122
NBD130
NBD137
NBD153
NBD155
NBD2
NBD4
NBD12
NBD27
NBD28
NBD36
NBD39
NBD58
NBD66
NBD72
NBD76
NBD85
NBD91
NBD95
NBD102
NBD132
NBD134
NBD136
NBD148
NBD149
NBD151
NBD154
E001 E002 E003 E004 E005 E006 E007 E008 E009 E010 E011 E012 E014 E015 E018 E019 E020 E021 E022 E024 E025 E026 E027 E028 E029 E030 E032 E033 E034 E035 E036 E039 E040 E041 E042 E043 E044
Color Key
and Histogram
Count
0
−4 −2 0 2 4
Column Z−Score
ALT ATRXmut
ALT ATRXwt
ATRX deletion
ATRX SNV/Indel/duplication
b
TMM group
C-Circle
Sex
Number of SNVs
Number of Indels
Number of SVs
Telomere content
37% PTPRD
30% NAV2
26% EYS
22% LRP2
19% SYNE1
19% UNC5C
15% LAMA3
11% BPTF
NBD91 NBD3 NBD72 NBD135 NBD148 NBD76 NBD66 NBD36 NBD12 NBD136 NBD151 NBD39 NBD41 NBD154 NBD58 NBD132 NBD118 NBD134 NBD149 NBD2 NBD102 NBD4 NBD27 NBD28 NBD85 NBD95 NBD123
ALT ATRXmut
ALT ATRXwt
MNA
TERT
PTPRD
NAV2
EYS
LRP2
SYNE1
UNC5C
LAMA3
BPTF
TMM group
ALT ATRXmut
ALT ATRXwt
C-Circle
positive
negative
Sex
male
female
Alterations
SNV_splicing
SNV_nonsynonymous
SNV_stopgain_stoploss
insertion_deletion
SV_intronic
SV_exonic
CNV_AMP
CNV_HomoDel
CNV_DEL
CNV_LOH
Telomere content
4
2
0
−2
−4
Number of SNVs
15000
10000
5000
0
Number of Indels
15000
10000
5000
0
Number of SVs
1500
1000
500
0
Frequency
100
80
60
40
20
0

Supplementary Figure 12: Exon specific expression of *ATRX* and most frequent mutations in ALT-positive *ATRX* wild-type neuroblastomas

(a) ATRX per exon normalized mRNA expression. mRNA expression (log2 RPKM) per exon (E1-E44) of *ATRX* in ALT neuroblastomas. Tumors with *ATRX* deletions are compared to tumors with other *ATRX* mutations and *ATRX* wild-type tumors. See Supplementary Table 4 for *ATRX* exon model. (b) Most recurrent mutations in ALT-positive *ATRX*wt neuroblastomas. Only genes with at least three exonic events (SVs, INDELs, SNVs) are shown. Exonic single nucleotide variations (SNVs), exonic small insertions and deletions (INDELs), structural variations in exonic and intronic regions (SVs) as well as copy number changes are illustrated. See top panel for telomere features and total number of called SNVs, SVs and INDELs. Frequency calculations on the left are based on SNVs, SVs, INDELs and CNVs. Black and white heatmap on the right side indicates the frequency of mutations in the respective gene in neuroblastoma groups of different telomere maintenance mechanisms. Copy number changes (deletion, LOH) were not considered for frequency calculations in the heatmap (right panel).

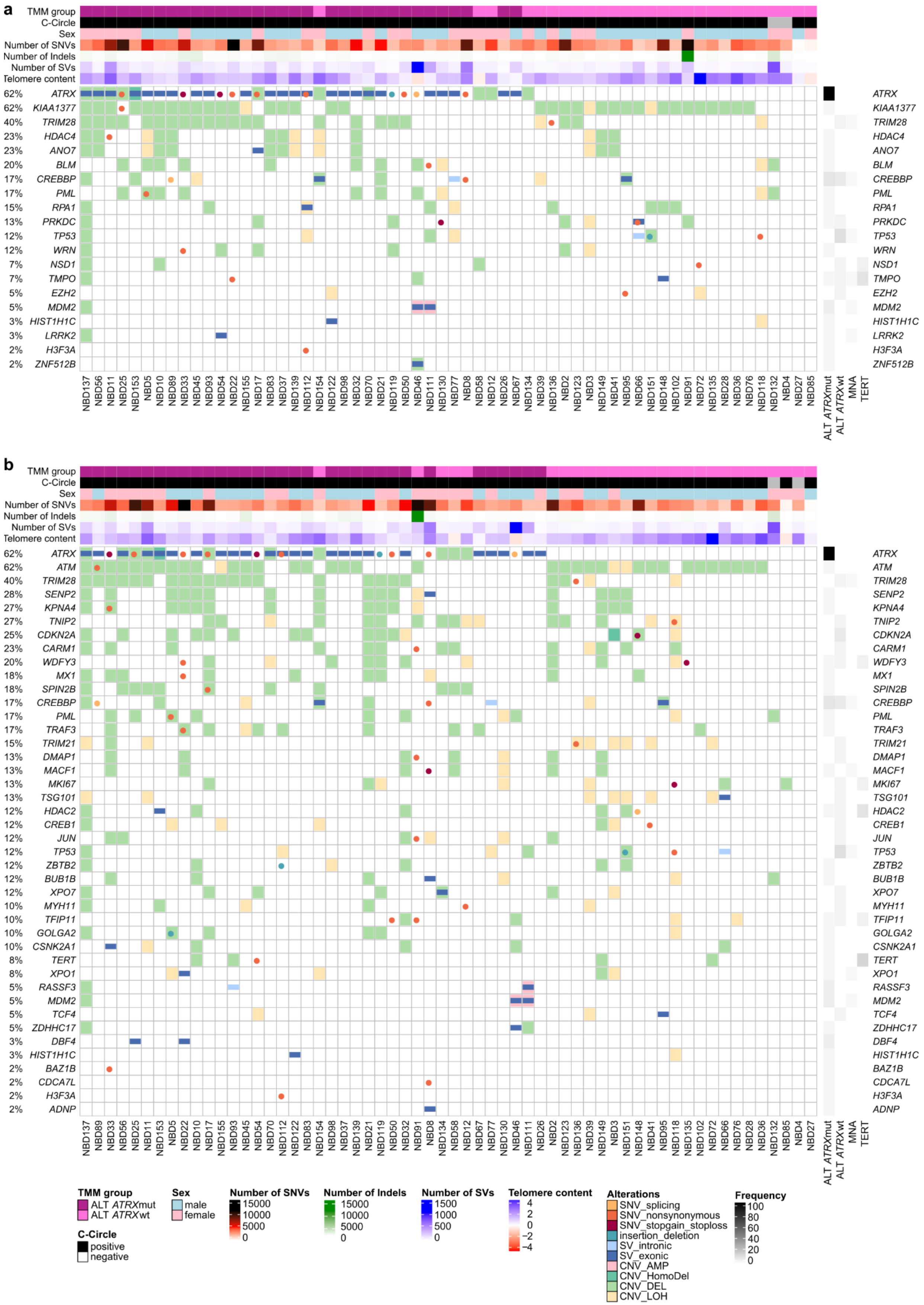
a
TMM group
C-Circle
Sex
Number of SNVs
Number of Indels
Number of SVs
Telomere content
62% ATRX
62% KIAA1377
40% TRIM28
23% HDAC4
23% ANO7
20% BLM
17% CREBBP
17% PML
15% RPA1
13% PRKDC
12% TP53
12% WRN
7% NSD1
7% TMPO
5% EZH2
5% MDM2
3% HIST1H1C
3% LRRK2
2% H3F3A
2% ZNF512B
NBD137 NBD56 NBD11 NBD25 NBD153 NBD5 NBD10 NBD89 NBD33 NBD45 NBD93 NBD54 NBD22 NBD155 NBD17 NBD83 NBD37 NBD139 NBD112 NBD154 NBD122 NBD98 NBD32 NBD70 NBD21 NBD119 NBD50 NBD46 NBD111 NBD130 NBD77 NBD8 NBD58 NBD12 NBD26 NBD67 NBD134 NBD39 NBD136 NBD2 NBD123 NBD3 NBD149 NBD41 NBD95 NBD66 NBD151 NBD148 NBD102 NBD91 NBD72 NBD135 NBD28 NBD36 NBD76 NBD118 NBD132 NBD4 NBD27 NBD85
ALT ATRXmut
ALT ATRXwt
MNA
TERT
b
TMM group
C-Circle
Sex
Number of SNVs
Number of Indels
Number of SVs
Telomere content
62% ATRX
62% ATM
40% TRIM28
28% SENP2
27% KPNA4
27% TNIP2
25% CDKN2A
23% CARM1
20% WDFY3
18% MX1
18% SPIN2B
17% CREBBP
17% PML
17% TRAF3
15% TRIM21
13% DMAP1
13% MACF1
13% MKI67
13% TSG101
12% HDAC2
12% CREB1
12% JUN
12% TP53
12% ZBTB2
12% BUB1B
12% XPO7
10% MYH11
10% TFIP11
10% GOLGA2
10% CSNK2A1
8% TERT
8% XPO1
5% RASSF3
5% MDM2
5% TCF4
5% ZDHHC17
3% DBF4
3% HIST1H1C
2% BAZ1B
2% CDCA7L
2% H3F3A
2% ADNP
NBD137 NBD89 NBD33 NBD56 NBD25 NBD11 NBD153 NBD5 NBD22 NBD10 NBD17 NBD155 NBD93 NBD45 NBD54 NBD70 NBD112 NBD122 NBD83 NBD154 NBD98 NBD37 NBD139 NBD21 NBD119 NBD50 NBD32 NBD91 NBD8 NBD134 NBD58 NBD12 NBD67 NBD77 NBD130 NBD46 NBD111 NBD26 NBD2 NBD123 NBD136 NBD39 NBD149 NBD3 NBD151 NBD148 NBD41 NBD95 NBD118 NBD135 NBD102 NBD72 NBD66 NBD76 NBD28 NBD36 NBD132 NBD85 NBD4 NBD27
ALT ATRXmut
ALT ATRXwt
MNA
TERT
TMM group
ALT ATRXmut
ALT ATRXwt
C-Circle
positive
negative
Sex
male
female
Number of SNVs
15000
10000
5000
0
Number of Indels
15000
10000
5000
0
Number of SVs
1500
1000
500
0
Telomere content
4
2
0
−2
−4
Alterations
SNV_splicing
SNV_nonsynonymous
SNV_stopgain_stoploss
insertion_deletion
SV_intronic
SV_exonic
CNV_AMP
CNV_HomoDel
CNV_DEL
CNV_LOH
Frequency
100
80
60
40
20
0

Supplementary Figure 13: Mutations in ATRX/DAXX interaction partners

Mutations of ATRX (a) and DAXX (b) interaction partners in ALT-positive neuroblastomas. ATRX and DAXX interaction partners were obtained from BioGRID [https://thebiogrid.org/]. Only genes with at least one exonic event (SVs, INDELs, SNVs) are shown. Exonic single nucleotide variations (SNVs), exonic small insertions and deletions (INDELs), structural variations in exonic and intronic regions (SVs) as well as copy number changes are illustrated. See top panel for telomere features and total number of called SNVs, SVs and INDELs. Frequency calculations on the left are based on SNVs, SVs, INDELSs and CNVs. Black and white heatmap on the right side indicates the frequency of mutations in the respective gene in neuroblastoma groups of different telomere maintenance mechanisms. Copy number changes (deletion, LOH) were not considered for frequency calculations in the heatmap (right panel).

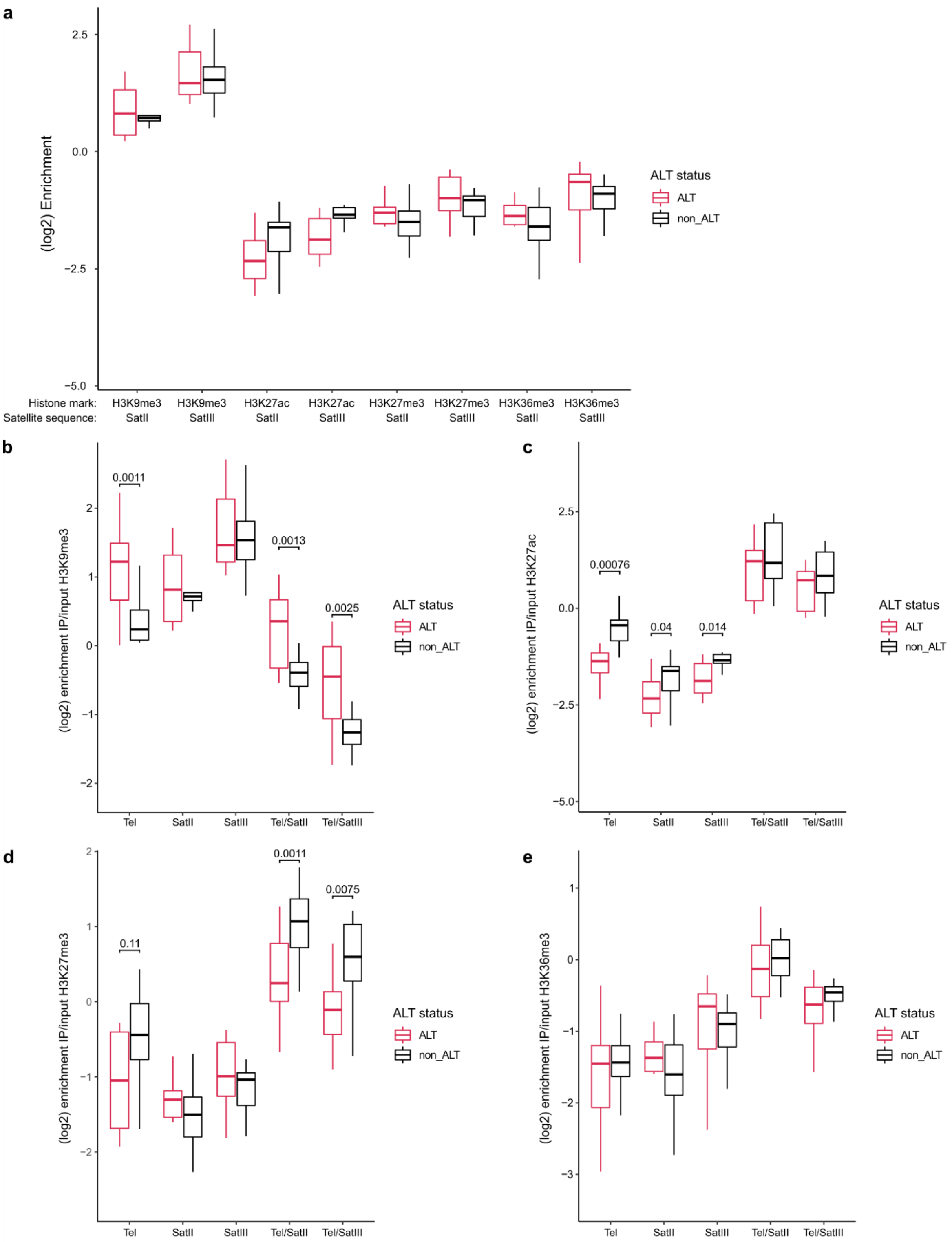

Supplementary Figure 14: Chromatin state at SatII and SatIII sequences
(a) log2 enrichment of ChIP signals relative to input signals of SatII and SatIII sequences for H3K9me3, H3K27ac, H3K27me3 and H3K36me3. ALT-positive tumors were compared to ALT-negative tumors (b-e) log2 enrichment in ChIP of H3K9me3 (b, *n* = 26), H3K27ac (c, *n* = 25), H3K27me3 (d, *n* = 25) and

H3K36me3 (e, *n* = 27) relative to input. Enrichment at telomeres, SatII and SatIII sequences is given. Additionally, telomeric signals were normalized to either SatII or SatIII. ALT-positive tumors are compared to ALT-negative tumors. Boxplots indicate the median value (middle line) and the 25th and 75th percentiles (box). The upper/lower whisker spans from the hinge to the largest/smallest value (values expanding a distance of 1.5 x inter quartile range are not considered). *n* describes the number of analyzed tumors. *P*-values were calculated using two-sided Wilcoxon rank sum tests.

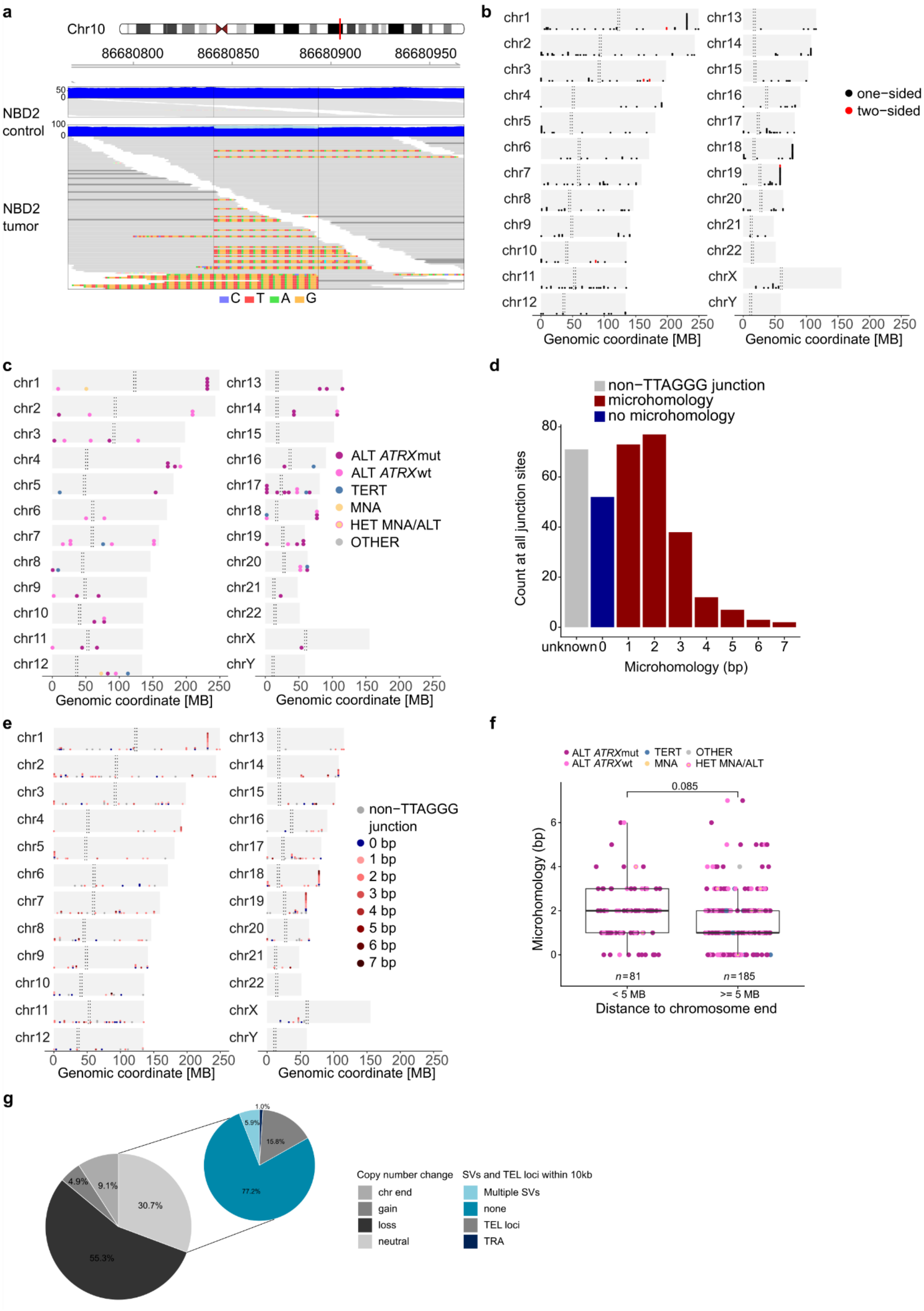
a
Chr10
86680800
86680850
86680900
86680950
NBD2 control
NBD2 tumor
C T A G
b
one−sided
two−sided
Genomic coordinate [MB]
c
ALT ATRXmut
ALT ATRXwt
TERT
MNA
HET MNA/ALT
OTHER
Genomic coordinate [MB]
d
non−TTAGGG junction
microhomology
no microhomology
Count at all junction sites
Microhomology (bp)
e
non−TTAGGG junction
0 bp
1 bp
2 bp
3 bp
4 bp
5 bp
6 bp
7 bp
Genomic coordinate [MB]
f
0.085
Microhomology (bp)
n=81
n=185
< 5 MB
>= 5 MB
Distance to chromosome end
g
Copy number change
chr end
gain
loss
neutral
SVs and TEL loci within 10kb
Multiple SVs
none
TEL loci
TRA

Supplementary Figure 15: Features of telomeric repeat loci

(a) Example of two telomeric repeat loci forming a two-sided or insertion-like event. Dark blue color indicates sequencing coverage and light blue color illustrates clipped sequences. Reads are shown in medium grey and clipped bases are color-coded. Non-telomeric ends of a discordant read pair are labeled in dark grey. (b) Chromosomal location of one-sided and two-sided telomeric repeat loci of the discovery cohort. (c) Chromosomal location of telomeric repeat loci in the INFORM cohort. (d) The occurrence of homologous bases (microhomology) between the reference genome and TTAGGG telomere repeats was counted for all telomeric repeat loci in the discovery cohort. (e) Degree of microhomology (color-coding) and chromosomal location for all telomeric repeat loci in the discovery cohort. (f) Degree of microhomology to TTAGGG of telomeric repeat loci being less than 5 MB or more than 5 MB away from the chromosome end. Boxplots indicate the median value (middle line) and the 25th and 75th percentiles (box). The upper/lower whisker spans from the hinge to the largest/smallest value (values expanding a distance of 1.5 x inter quartile range are not considered). Dots represent individual telomeric repeat loci. *n* gives the number of telomeric repeat loci in each group. *P*-value was calculated using a two-sided Wilcoxon rank sum test. (g) (left pie) Percentage of telomeric repeat loci overlapping with a copy number variation (events in group “chr end” are too close to the telomere and thus no CNV information could be obtained). (right pie) Percentage of copy number neutral telomeric repeat loci overlapping with an SV breakpoint within a 10 kb window (TRA = translocation, TEL loci = another telomeric repeat locus, Multiple SVs = more than one SV in 10 kb distance, none = no SV in 10kb window).

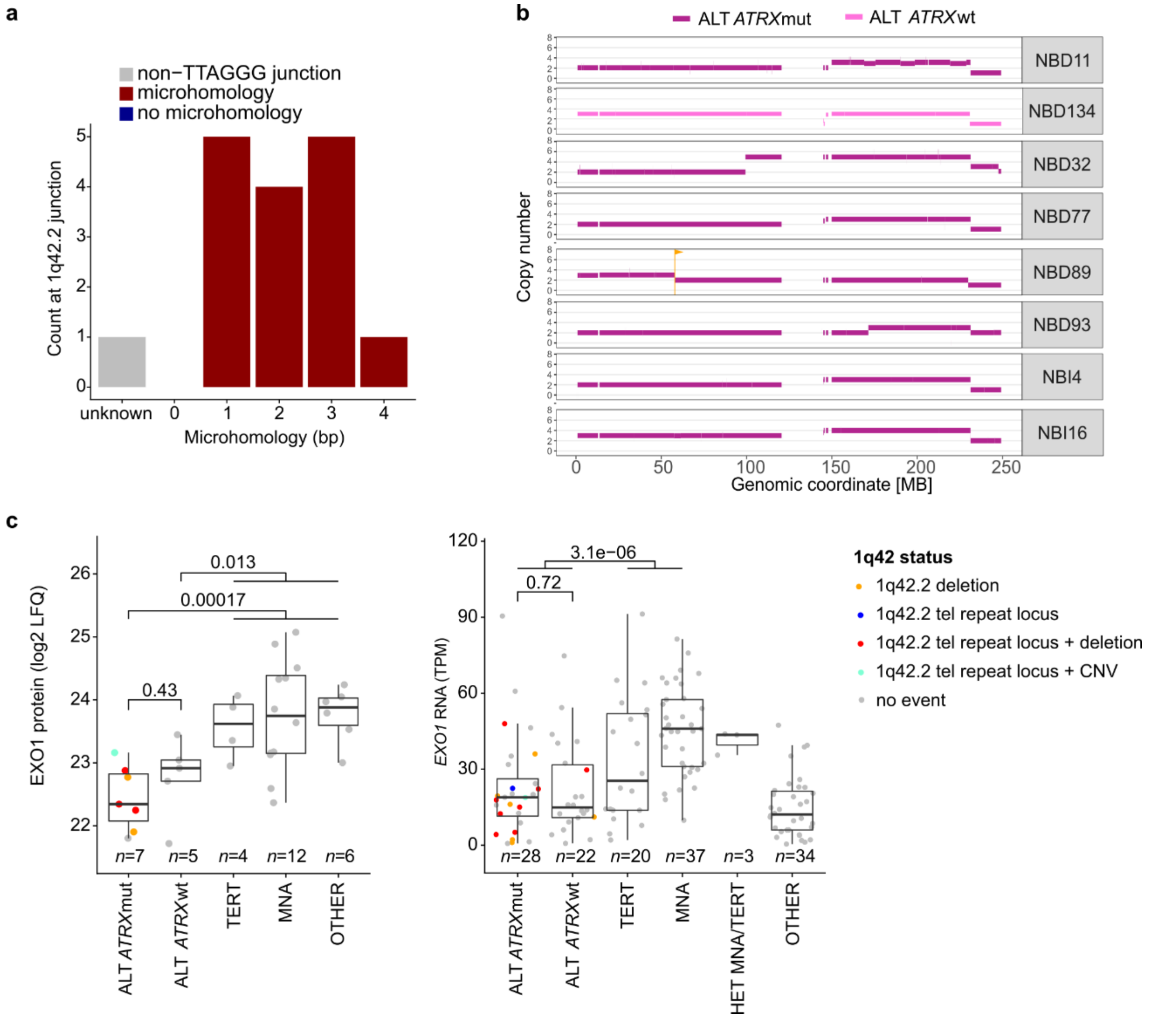

Supplementary Figure 16: 1q42.2-1qter deletion

(a) The occurrence of homologous bases between the reference genome and TTAGGG telomere repeats was counted for all telomeric repeat loci at the 1q42.2 hotspot. 1q42.2 telomeric repeat loci of NBD25 were excluded as they had a different directionality. (b) Chr1 copy number profile of tumors with a 1q42.2-1qter deletion without a detected telomeric repeat locus color coded by subgroup. chr1q42.2-1qter deletion was defined as a reduced copy number compared to the upstream neighboring segment. (c) Boxplots of protein and mRNA expression of *EXO1* in ALT *ATRX*-mutated and wild-type tumors compared to TERT, MNA and tumors without a telomere maintenance mechanism (OTHER). Colors indicate the presence of a telomeric repeat locus on chr1q42.2 and/or presence of a chr1q42.2-1qter deletion (compared to the neighboring upstream segment). Boxplots indicate the median value (middle line) and the 25th and 75th percentiles (box). The upper/lower whisker spans from the hinge to the largest/smallest value (values expanding a distance of 1.5 x inter quartile range are not considered). Dots represent individual tumors. *n* describes the number of analyzed tumors. *P* values were calculated with two-sided Wilcoxon rank sum tests.

II. Supplementary Tables

Supplementary Table 1: Two-sided telomeric repeat loci

ID	Chr	Junction 1[a]	Junction 2[a]	Distance[b]	Supporting read pairs[c]
NBD11	3	171927766	171927838	72	0
NBD22	19	57621777	57622140	363	0
NBD5	3	162504712	162506720	2008	10
NBD130	1	198923306	198923309	3	2
NBD2	10	86680841	86680894	53	0

[a] Chromosomal position of junction sites of contributing telomeric repeat loci
[b] Distance between the two junction sites
[c] Number of read pairs supporting an insertion like event, meaning that mates of a read pair map to both sides of the event

Supplementary Table 2: siRNA sequences

siRNAs	Sequence/ siRNA ID	Supplier
ATRX si1 (sh589)[a]	5'-GCAGATTGATATGAGAGGAAT-3'	Ambion Silencer™ select siRNA (custom)
ATRX si2 (sh590)[a]	5'-CGACAGAAACTAACCCTGTAA-3'	Ambion Silencer™ select siRNA (custom)
ATRX si3 (sh592)[a]	5'-CCGGTGGTGAACATAAGAAAT-3'	Ambion Silencer™ select siRNA (custom)
DAXX si1	s3935	Ambion Silencer™ select siRNA
DAXX si2	s3936	Ambion Silencer™ select siRNA
DAXX si3	s3937	Ambion Silencer™ select siRNA
Negative Control No. 1	4390843	Ambion Silencer™ select siRNA
Negative Control No. 2	4390846	Ambion Silencer™ select siRNA

[a] ATRX siRNA target sequences were taken from[2] and obtained from Thermo Fisher as custom silencer select siRNA

Supplementary Table 3: Antibodies

Target	Species	Clone	Supplier	Dilution	Order No.	Lot. No.
ATRX	rabbit	polyclonal	Sigma	1:1000	HPA001906	CI118688
DAXX	rabbit	monoclonal E94	Abcam	1:5000	ab32140	GR155454-9
Vinculin-HRP	mouse	monoclonal 7F9	Santa Cruz	1:1000	sc73614	A2319
Secondary rabbit-HRP	goat	polyclonal	Dianova	1:2000	111-035-144	134896
H3K27me3	rabbit	polyclonal	Active Motif	3µg/IP	39155	31014017
H3K36me3	rabbit l	polyclonal	Abcam	3µg/IP	ab9050	GR273250-1
H3K9me3	rabbit	polyclonal	Abcam	3µg/IP	ab8898	GR148830-2
H3K27ac	rabbit	polyclonal	Abcam	3µg/IP	ab4729	GR183919-2
anti-dig-FITC antibody	sheep	polyclonal	Roche	1:100	11207741910	11404500

Supplementary Table 4: DEXseq *ATRX* exon model

Exon	ID	Chr	Start	End	Width	Strand
ENSG00000085224.16:E001	E001	chrX	76760356	76760377	22	-
ENSG00000085224.16:E002	E002	chrX	76760378	76764107	3730	-
ENSG00000085224.16:E003	E003	chrX	76776266	76776394	129	-
ENSG00000085224.16:E004	E004	chrX	76776881	76776976	96	-
ENSG00000085224.16:E005	E005	chrX	76777741	76777866	126	-
ENSG00000085224.16:E006	E006	chrX	76778730	76778879	150	-
ENSG00000085224.16:E007	E007	chrX	76812922	76813116	195	-
ENSG00000085224.16:E008	E008	chrX	76814140	76814317	178	-
ENSG00000085224.16:E009	E009	chrX	76829715	76829823	109	-
ENSG00000085224.16:E010	E010	chrX	76845304	76845410	107	-
ENSG00000085224.16:E011	E011	chrX	76849166	76849319	154	-
ENSG00000085224.16:E012	E012	chrX	76854880	76855049	170	-
ENSG00000085224.16:E013	E013	chrX	76855201	76855203	3	-
ENSG00000085224.16:E014	E014	chrX	76855204	76855289	86	-
ENSG00000085224.16:E015	E015	chrX	76855903	76856033	131	-
ENSG00000085224.16:E016	E016	chrX	76856034	76856224	191	-
ENSG00000085224.16:E017	E017	chrX	76871720	76871979	260	-
ENSG00000085224.16:E018	E018	chrX	76872081	76872198	118	-
ENSG00000085224.16:E019	E019	chrX	76874274	76874449	176	-
ENSG00000085224.16:E020	E020	chrX	76875863	76876000	138	-
ENSG00000085224.16:E021	E021	chrX	76888695	76888872	178	-
ENSG00000085224.16:E022	E022	chrX	76889054	76889200	147	-
ENSG00000085224.16:E023	E023	chrX	76889913	76890084	172	-
ENSG00000085224.16:E024	E024	chrX	76890085	76890194	110	-
ENSG00000085224.16:E025	E025	chrX	76891406	76891547	142	-
ENSG00000085224.16:E026	E026	chrX	76907604	76907843	240	-
ENSG00000085224.16:E027	E027	chrX	76909588	76909690	103	-
ENSG00000085224.16:E028	E028	chrX	76912050	76912143	94	-
ENSG00000085224.16:E029	E029	chrX	76918871	76919047	177	-
ENSG00000085224.16:E030	E030	chrX	76920134	76920267	134	-
ENSG00000085224.16:E031	E031	chrX	76931535	76931720	186	-
ENSG00000085224.16:E032	E032	chrX	76931721	76931793	73	-
ENSG00000085224.16:E033	E033	chrX	76937012	76937093	82	-
ENSG00000085224.16:E034	E034	chrX	76937094	76940085	2992	-
ENSG00000085224.16:E035	E035	chrX	76940431	76940498	68	-
ENSG00000085224.16:E036	E036	chrX	76944311	76944420	*110*	-
ENSG00000085224.16:E037	E037	chrX	76946296	76946418	123	-
ENSG00000085224.16:E038	E038	chrX	76949188	76949312	125	-
ENSG00000085224.16:E039	E039	chrX	76949313	76949426	114	-
ENSG00000085224.16:E040	E040	chrX	76952065	76952192	128	-
ENSG00000085224.16:E041	E041	chrX	76953071	76953123	53	-
ENSG00000085224.16:E042	E042	chrX	76954062	76954117	56	-
ENSG00000085224.16:E043	E043	chrX	76972608	76972720	*113*	-
ENSG00000085224.16:E044	E044	chrX	77041468	77041702	235	-

III. Supplementary References

1 Braun, D. M., Chung, I., Kepper, N., Deeg, K. I. & Rippe, K. TelNet - a database for human and yeast genes involved in telomere maintenance. *BMC Genet* **19**, 32, doi:10.1186/s12863-018-0617-8 (2018).

2 Lovejoy, C. A. *et al.* Loss of ATRX, genome instability, and an altered DNA damage response are hallmarks of the alternative lengthening of telomeres pathway. *PLoS Genet* **8**, e1002772, doi:10.1371/journal.pgen.1002772 (2012).
