# Supplementary material for: Alternative lengthening of telomeres in childhood neuroblastoma from genome to proteome
Source: Nat Commun. 2021 Feb 24;12:1269. doi: 10.1038/s41467-021-21247-8 (PMC7904810; doi:10.1038/s41467-021-21247-8)
Supplement: Supplementary file 3 — Description of Additional Supplementary Files [file 41467_2021_21247_MOESM3_ESM.pdf]

Description of Additional Supplementary Files

Supplementary Data 1: Clinical information for tumors in the screening cohort
C-Circle status and C-Circle intensity as well as clinical parameters including INSS stage, age, treatment protocol, disease period, sex, NB2004 risk status (HR = high-risk, MRG = intermediate risk, observation = low risk) and *MYCN* status (assessed as routine diagnostic marker; amp = amplified, normal = normal copy number, n.e. = inconclusive) are given.

Supplementary Data 2: Clinical information and available data types for tumors in the INFORM cohort
C-Circle status, telomere content, TMM subgrouping, *TERT* and *ATRX* status are indicated. Clinical parameters including INSS stage, age, treatment protocol, disease period, sex, NB2004 risk status (HR = high-risk, MRG = intermediate risk, observation = low risk) and *MYCN* status (assessed as routine diagnostic marker; amp = amplified, normal = normal copy number, n.e. = inconclusive) are given. Available data types (lcWGS, hcWGS, WES) are indicated.

Supplementary Data 3: Clinical information and available data types for tumors in the discovery cohort
C-Circle status, telomere content, TMM subgrouping, *TERT* and *ATRX* status are indicated. Presence of a telomeric repeat locus on chr1q42.2 and presence of a 1q42-1qter deletion are given. Event free survival (EFS) and overall survival (OS) data, as well as clinical parameters including INSS stage, age, treatment protocol, disease period, sex, NB2004 risk status (HR = high-risk, MRG = intermediate risk, observation = low risk) and *MYCN* status (assessed as routine diagnostic marker; amp = amplified, normal = normal copy number, n.e. = inconclusive) are given. Available data types (WGS, RNA-sequencing, ChIP-sequencing and proteome analysis) are indicated. Tumor ploidy and purity were assessed based on the sequencing results.

Supplementary Data 4: Genomic coordinates of *ATRX* mutations
List of identified *ATRX* mutations in tumors of the discovery and INFORM cohort. Type of mutation and exact genomic position is given.

Supplementary Data 5: List of 470 differentially expressed proteins between ALT-positive and ALT-negative neuroblastomas
Protein expression determined using label free quantification (LFQ). P values were calculated using a two-sided Student's t-test. Differential expression was defined as $P \leq 0.01$; fold change ≥ 2. Proteins being associated with telomere maintenance and biology as well as proteins being regulated by MYCN are marked.

Supplementary Data 6: Enriched UniProt keywords with significant enrichments in ALT-positive tumors on mRNA and protein level

UniProt keywords ([https://www.uniprot.org/keywords/]; retrieve on 2016-11-02) with significant enrichments (BH adjusted $P < 0.000005$, at least 2.5-fold enriched, at least 3 protein/mRNAs per keyword) in at least one group in significantly more or less abundant mRNA or proteins comparing each group to all other samples (BH adjusted $P < 0.05$). Enrichment P values were calculated by hypergeometric test and multiple-testing adjusted by Benjamini-Hochberg method.

Supplementary Data 7: SNVs and INDELs in discovery cohort

All functional SNVs and INDELs are listed for the individual tumors of the discovery cohort. Exact chromosomal location and type of alteration is indicated.

Supplementary Data 8: SVs in discovery cohort

All SVs for the individual tumors of the discovery cohort are listed. Exact chromosomal location and type of alteration is indicated.

Supplementary Data 9: Amplifications and homozygous deletions in discovery cohort

List of all amplifications and homozygous deletions affecting genes for the individual tumors of the discovery cohort. (amp = amplification, HOMODEL = homozygous deletion)
