# Supplementary material for: Alternative lengthening of telomeres in childhood neuroblastoma from genome to proteome
Source: Nat Commun. 2021 Feb 24;12:1269. doi: 10.1038/s41467-021-21247-8 (PMC7904810; doi:10.1038/s41467-021-21247-8)
Supplement: Supplementary file 13 — Reporting Summary [file 41467_2021_21247_MOESM13_ESM.pdf]

nature research

Corresponding author(s): PD Dr. Frank Westermann

Last updated by author(s): 10.12.2020

Reporting Summary

Nature Research wishes to improve the reproducibility of the work that we publish. This form provides structure for consistency and transparency in reporting. For further information on Nature Research policies, see our Editorial Policies and the Editorial Policy Checklist.

Statistics

For all statistical analyses, confirm that the following items are present in the figure legend, table legend, main text, or Methods section.

n/a	Confirmed	
☐	☒	The exact sample size (*n*) for each experimental group/condition, given as a discrete number and unit of measurement
☐	☒	A statement on whether measurements were taken from distinct samples or whether the same sample was measured repeatedly
☐	☒	The statistical test(s) used AND whether they are one- or two-sided<br>*Only common tests should be described solely by name; describe more complex techniques in the Methods section.*
☐	☒	A description of all covariates tested
☐	☒	A description of any assumptions or corrections, such as tests of normality and adjustment for multiple comparisons
☐	☒	A full description of the statistical parameters including central tendency (e.g. means) or other basic estimates (e.g. regression coefficient) AND variation (e.g. standard deviation) or associated estimates of uncertainty (e.g. confidence intervals)
☐	☒	For null hypothesis testing, the test statistic (e.g. *F*, *t*, *r*) with confidence intervals, effect sizes, degrees of freedom and *P* value noted<br>*Give P values as exact values whenever suitable.*
☒	☐	For Bayesian analysis, information on the choice of priors and Markov chain Monte Carlo settings
☒	☐	For hierarchical and complex designs, identification of the appropriate level for tests and full reporting of outcomes
☒	☐	Estimates of effect sizes (e.g. Cohen's *d*, Pearson's *r*), indicating how they were calculated

Our web collection on statistics for biologists contains articles on many of the points above.

Software and code

Policy information about availability of computer code

Data collection	-ISIS software version 5.7.4<br>-Cytovision software version 7.6<br>-Leica CW 4000 FISH software
Data analysis	Software used in the study:<br>-ImageJ version 1.51<br>-BWA-MEM version 0.7.8, 0.7.8-r2.08 or 0.7.15<br>-Sambamba version 0.6.5<br>-Samtools version 0.1.19<br>-ACEseq version 1.2.8<br>-SOPHIA version 1.0.16 or 1.2.16<br>-TelomereHunter version 1.0.1<br>-STAR Version 2.5.2b<br>-STAR version 2.4.1<br>-SAM tools version 1.3.1<br>-DEXSeq version 1.24.3<br>-featurecounts version 1.5.1<br>-DESeq2 version 1.18.1<br>-MACS2 version 2.1<br>-deepTools2 suite version 3.0<br>-Trimgalore tool version 0.4<br>-Bowtie2 version 2.3<br>-SICER version 1.1<br>-MaxQuant software version 1.5.5.1

-Perseus software version 1.5.5.3
-R base version 3.5.1 or 3.6.1
-R ggplot2 version 3.2.0 or version 3.3.1
-R ggpubr version 0.3.0
-R survminer version 0.4.7

We have provided a code availability section as part of the methods:
We have described the algorithms and software availability as part of the respective methods section. TelomereHunter v1.0.1 is available at [https://www.dkfz.de/en/applied-bioinformatics/telomerehunter/telomerehunter.html]. The pipeline used to detect telomeric repeat loci is available via Github [https://github.com/linasieverling/TelomereRepeatLoci]. The workflow used for RNA-sequencing is available at [https://github.com/DKFZ-ODCF/RNAseqWorkflow]. SV calling was done using the workflow available at [https://github.com/DKFZ-ODCF/SophiaWorkflow]. The SNV calling and Indel workflow is available at [https://github.com/DKFZ-ODCF/SNVCallingWorkflow] and [https://github.com/DKFZ-ODCF/IndelCallingWorkflow]. The ACEseq workflow is available at [https://github.com/DKFZ-ODCF/ACEseqWorkflow]. ChIP-sequencing workflow is available at [https://github.com/hdsu-bioquant/chipseq_telomeres/].

For manuscripts utilizing custom algorithms or software that are central to the research but not yet described in published literature, software must be made available to editors and reviewers. We strongly encourage code deposition in a community repository (e.g. GitHub). See the Nature Research guidelines for submitting code & software for further information.

Data

Policy information about availability of data

All manuscripts must include a data availability statement. This statement should provide the following information, where applicable:

- Accession codes, unique identifiers, or web links for publicly available datasets
- A list of figures that have associated raw data
- A description of any restrictions on data availability

All high-throughput sequencing data and proteome data has been archived at the European Genome-phenome Archive (https://www.ebi.ac.uk/ega/) and can be found under accession number EGAS00001004349 [https://www.ebi.ac.uk/ega/studies/EGAS00001004349]. Part of the whole genome and RNA sequencing data was previously published (Peifer at al., Nature 2015) and is available under the accession number EGAS00001001308 [https://www.ebi.ac.uk/ega/studies/EGAS00001001308]. The data is deposited under controlled access. Access can be obtained by contacting FW. Access to data requires a data transfer agreement. mRNA and protein expression data are available as datasets in the R2 database and can be interactively analyzed. mRNA expression data can be found under the name “Tumor Neuroblastoma ALT - Westermann - 144 - tpm - gencode19” [https://hgserver1.amc.nl/cgi-bin/r2/main.cgi?table=ps_avgpres_2010fwr144_gencode19]. Protein data can be found under the name “Tumor Neuoblastoma ALT (Protein) – Westermann – 34 – LFQ - fw2010prot” [https://hgserver1.amc.nl/cgi-bin/r2/main.cgi?table=ps_avgpres_fw2010prot34_fw2010prot]. All functional SNVs, INDELs, SVs and copy number alterations (only amplification and homozygous deletions) for all tumors in the discovery cohort are provided as Supplementary Data 7-9. Source data are provided with this paper. The remaining data are available within the Article, Supplementary Information or available from the authors upon request.
Protein sequence information was extracted from the human Uniprot database ([https://www.uniprot.org/]; release 2014-10) and contaminants database included in the MaxQuant software. Associations with telomere maintenance and biology were extracted from the Telnet database [https://malone2.bioquant.uni-heidelberg.de/fmi/webd/TelNet]. ATRX and DAXX interaction partners were extracted from the BioGRID database [https://thebiogrid.org/].

Field-specific reporting

Please select the one below that is the best fit for your research. If you are not sure, read the appropriate sections before making your selection.

☒ Life sciences ☐ Behavioural & social sciences ☐ Ecological, evolutionary & environmental sciences

For a reference copy of the document with all sections, see nature.com/documents/nr-reporting-summary-flat.pdf

Life sciences study design

All studies must disclose on these points even when the disclosure is negative.

Sample size	No statistical sample size calculations were performed. C-Circle screening was done on all available tumor tissues from the Neuroblastoma Biobank Heidelberg. Since the primary aim of the study was to characterize as many ALT-positive samples as possible, all ALT-positive samples with sufficient available material and quality of material were further characterized. Data of additional ALT-negative tumors was collected as part of a previously published study (Peifer et al., 2015) and other sequencing projects on high risk neuroblastoma tumors (e.g. HIPO2). The discovery cohort is not representative with regards to frequencies of individual tumor subtypes in the clinics. However, the discovery cohort reflects different types of telomere maintenance mechanisms and molecular backgrounds.<br>The INFORM cohort comprises all enrolled neuroblastoma patients at relapse between 2015 and 2018.
Data exclusions	For ChIP-sequencing analysis samples with a fraction of reads in peaks (FRIP) < 1% were excluded, which is in line with the guidelines of the ENCODE consortium (Landt et al., Genome Res 2012).
Replication	All tumor tissues were analyzed once. All experimental analysis was done in three biological replicates.
Randomization	There was no randomization done for the analysis. Tumor samples were retrospectively grouped based on genetic evidence fitting to a telomere maintenance subgroup.
Blinding	This is a retrospective study on neuroblastoma telomere maintenance. Patients were not grouped to experimental groups. Tumors were grouped into genetic telomere maintenance groups to retrospectively assess prognostic impact and potential impact on risk stratification of these genetic markers. C-Circle screening was used to determine the ALT-status and to enrich the discovery cohort for ALT-positive tumors.

The Investigators were blind when performing C-Circle screening.

Reporting for specific materials, systems and methods

We require information from authors about some types of materials, experimental systems and methods used in many studies. Here, indicate whether each material, system or method listed is relevant to your study. If you are not sure if a list item applies to your research, read the appropriate section before selecting a response.

Materials & experimental systems

n/a	Involved in the study
☐	☒ Antibodies
☐	☒ Eukaryotic cell lines
☒	☐ Palaeontology and archaeology
☒	☐ Animals and other organisms
☐	☒ Human research participants
☐	☒ Clinical data
☒	☐ Dual use research of concern

Methods

n/a	Involved in the study
☐	☒ ChIP-seq
☐	☒ Flow cytometry
☒	☐ MRI-based neuroimaging

Antibodies

Antibodies used

Western blot:
ATRX; rabbit polyclonal; Sigma HPA001906; Lot.CI118688; dilution 1:1000
DAXX; rabbit monoclonal [E94]; Abcam ab32140; Lot.GR155454-9; dilution 1:5000
Vinculin-HRP; mouse monoclonal 7F9; Santa Cruz sc73614; Lot.A2319; dilution 1:1000
Secondary rabbit-HRP; goat polyclonal; Dianova 111-035-144; Lot. 134896; dilution 1:2000
FISH:
anti-dig-FITC antibody; sheep polyclonal; Roche 11207741910; Lot.11404500; dilution 1:100
ChIP-seq: For all ChIP-seq experiments 3µg of antibody were used per ChIP.
H3K27me3; rabbit polyclonal; Active Motif 39155; Lot.31014017
H3K36me3; rabbit polyclonal; Abcam ab9050; Lot.GR273250-1
H3K9me3; rabbit polyclonal; Abcam; ab8898; Lot.GR148830-2
H3K27ac; rabbit polyclonal; Abcam; ab4729; Lot.GR183919-2

Validation

A protein size marker was run on every western blot and the size of the assessed bands was compared to the manufactures information. For ATRX and DAXX siRNA knockdowns were also used to evaluate the antibodies.
ATRX antibody is specific to human ATRX protein and is part of the prestige antibody line and was additionally validated using siRNA knockdown and orthogonal RNAseq experiments. See description https://www.sigmaaldrich.com/technical-documents/articles/biology/antibody-enhanced-validation.html. DAXX antibody was knockout validated by the manufacturer, is recombinantly produced and only reactive to human DAXX.
ATRX and DAXX antibodies are validated for the use in western blotting by the manufacturer. All ChIP-seq antibodies are validated for the use in ChIP-seq by the manufacturer.

Eukaryotic cell lines

Policy information about cell lines

Cell line source(s)

CHLA-90, NBL-S, SK-N-BE2 were privided by Dr. Larissa Savelyeva (German Cancer Research Center)

Authentication

Cell line authentication was done using the Muliplex cell line authentication test (MCA) by MULITPLEXION. https://www.multiplexion.de/en/multiplex-human-cell-line-authentication

Mycoplasma contamination

Testing for Mycoplasma contamination was done using the Muliplex cell line contamination test (MCCT) by MULTIPLEXION. https://www.multiplexion.de/en/cell-contamination-test. All cell lines were tested negative for Mycoplasma contamination.

Commonly misidentified lines
(See ICLAC register)

No commonly misidentified cell lines were used.

Human research participants

Policy information about studies involving human research participants

Population characteristics

Clinical parameters for all patients of the Screening, Discovery cohort and INFORM cohort are given Supplementary Data 1-3. All children were enrolled in Germany (small subset in Austria).

Recruitment

Patients of the Screening/Discovery cohort were enrolled in the Neuroblastoma clinical trials of the GPOH (Gesellschaft für Pädiatrische Onkologie und Hämatologie) between 1991 and 2018. Patients at relapse were enrolled in the INFORM registry trial between 2015 and 2018. For INFORM, only patients being enrolled in Germany were analysed. Tumor material of these patients was analyzed retrospectively based on availability of material. ALT-positive tumors were artificially enriched in the

	discovery cohort to study the molecular characteristics of this subgroup in more detail. This was necessary since ALT-positive neuroblastomas are relatively rare in absolute numbers, especially in the primary disease period. The C-Circle screening cohort contains a large proportion of low risk tumors. Low risk tumors are enriched, since neuroblastomas were routinely screened for by measuring catecholamines in the urin of children in Germany.
Ethics oversight	All neuroblastoma trials were approved by the Ethics Committee of the Medical Faculty, University of Cologne. INFORM registry trial was approved by Ethics Committee of the University of Heidelberg. All patients or their parents signed an informed consent.

Note that full information on the approval of the study protocol must also be provided in the manuscript.

Clinical data

Policy information about clinical studies

All manuscripts should comply with the ICMJE guidelines for publication of clinical research and a completed CONSORT checklist must be included with all submissions.

Clinical trial registration	This is a retrospective analysis of tumor material from patients being enrolled in the neuroblastoma trials of the GPOH and/or INFORM registry trial. Minimal clinical information (age, stage, risk markers) and survival data was provided by the Neuroblastoma trial office. This clinical data was used to evaluate retrospectively the prognostic impact of certain molecular markers and to study the importance of this markers for risk assessment and allocation to future clinical trials.
Study protocol	Not applicable. This is a retrospective analysis of tumor material from patients being enrolled in the neuroblastoma trials of the GPOH and/or INFORM registry trial.
Data collection	Not applicable. This is a retrospective analysis of tumor material from patients being enrolled in the neuroblastoma trials of the GPOH and/or INFORM registry trial.
Outcomes	Not applicable. This is a retrospective analysis of tumor material from patients being enrolled in the neuroblastoma trials of the GPOH and/or INFORM registry trial.

ChIP-seq

Data deposition

☒ Confirm that both raw and final processed data have been deposited in a public database such as GEO.

☒ Confirm that you have deposited or provided access to graph files (e.g. BED files) for the called peaks.

Data access links<br>*May remain private before publication.*	EGAS00001004349
Files in database submission	Fastq files for H3K27me3 (n=25 tumors) , H3K36me3 (n=27 tumors), H3K9me3 (n=26 tumors), H3K27ac (n=25) deposited under EGAS00001004349, number of extracted Tel, SatII and SatIII reads given in source data file.
Genome browser session<br>(e.g. UCSC)	no longer applicable

Methodology

Replicates	Each tumor was analyzed once.
Sequencing depth	ChIP-sequencing was done using Illumina HiSeq2000 50 bp single end sequencing.
Antibodies	H3K27me3 (39155, Active Motif), H3K36me3 (ab9050, Abcam), H3K9me3 (ab8898, Abcam), H3K27ac (ab4729, Abcam)<br>3 ug of each antibody were used per IP.
Peak calling parameters	Peaks were called using MACS2 (https://github.com/taoliu/MACS) for H3K27ac or SICER for H3K9me3, H3K27me3 and H3K36me3.
Data quality	Samples with a fraction of reads in peaks <1 were excluded. , which is in line with the guidelines of the ENCODE consortium (Landt et al., Genome Res 2012).
Software	CHIP-sequencing data analysis is described in the methods section. ChIP-sequencing workflow is available at [https://github.com/hdsu-bioquant/chipseq_telomeres/].

Flow Cytometry

Plots

Confirm that:

- [ ] The axis labels state the marker and fluorochrome used (e.g. CD4-FITC).
- [ ] The axis scales are clearly visible. Include numbers along axes only for bottom left plot of group (a 'group' is an analysis of identical markers).
- [ ] All plots are contour plots with outliers or pseudocolor plots.
- [ ] A numerical value for number of cells or percentage (with statistics) is provided.

Methodology

Sample preparation	FACS analysis of neuroblastoma tumors was done as part of a previously published study (Ryl et al. Cell Syst 2017)
Instrument	*Identify the instrument used for data collection, specifying make and model number.*
Software	*Describe the software used to collect and analyze the flow cytometry data. For custom code that has been deposited into a community repository, provide accession details.*
Cell population abundance	*Describe the abundance of the relevant cell populations within post-sort fractions, providing details on the purity of the samples and how it was determined.*
Gating strategy	*Describe the gating strategy used for all relevant experiments, specifying the preliminary FSC/SSC gates of the starting cell population, indicating where boundaries between "positive" and "negative" staining cell populations are defined.*

- [ ] Tick this box to confirm that a figure exemplifying the gating strategy is provided in the Supplementary Information.
